# Characterising and justifying sample size sufficiency in interview-based studies: systematic analysis of qualitative health research over a 15-year period

**DOI:** 10.1186/s12874-018-0594-7

**Published:** 2018-11-21

**Authors:** Konstantina Vasileiou, Julie Barnett, Susan Thorpe, Terry Young

**Affiliations:** 1 Department of Psychology, University of Bath, Building 10 West, Claverton Down, Bath, BA2 7AY UK; 2 School of Psychology, Newcastle University, Ridley Building 1, Queen Victoria Road, Newcastle upon Tyne, NE1 7RU UK; 3 Department of Computer Science, Brunel University London, Wilfred Brown Building 108, Uxbridge, UB8 3PH UK

**Keywords:** Sample size, Sample size justification, Sample size characterisation, Data adequacy, Qualitative health research, Qualitative interviews, Review, Systematic analysis

## Abstract

**Background:**

Choosing a suitable sample size in qualitative research is an area of conceptual debate and practical uncertainty. That sample size principles, guidelines and tools have been developed to enable researchers to set, and justify the acceptability of, their sample size is an indication that the issue constitutes an important marker of the quality of qualitative research. Nevertheless, research shows that sample size sufficiency reporting is often poor, if not absent, across a range of disciplinary fields.

**Methods:**

A systematic analysis of single-interview-per-participant designs within three health-related journals from the disciplines of psychology, sociology and medicine, over a 15-year period, was conducted to examine whether and how sample sizes were justified and how sample size was characterised and discussed by authors. Data pertinent to sample size were extracted and analysed using qualitative and quantitative analytic techniques.

**Results:**

Our findings demonstrate that provision of sample size justifications in qualitative health research is limited; is not contingent on the number of interviews; and relates to the journal of publication. Defence of sample size was most frequently supported across all three journals with reference to the principle of saturation and to pragmatic considerations. Qualitative sample sizes were predominantly – and often without justification – characterised as insufficient (i.e., ‘small’) and discussed in the context of study limitations. Sample size insufficiency was seen to threaten the validity and generalizability of studies’ results, with the latter being frequently conceived in nomothetic terms.

**Conclusions:**

We recommend, firstly, that qualitative health researchers be more transparent about evaluations of their sample size sufficiency, situating these within broader and more encompassing assessments of *data adequacy*. Secondly, we invite researchers critically to consider how saturation parameters found in prior methodological studies and sample size community norms might best inform, and apply to, their own project and encourage that data adequacy is best appraised with reference to features that are *intrinsic* to the study at hand. Finally, those reviewing papers have a vital role in supporting and encouraging transparent study-specific reporting.

**Electronic supplementary material:**

The online version of this article (10.1186/s12874-018-0594-7) contains supplementary material, which is available to authorized users.

## Background

Sample adequacy in qualitative inquiry pertains to the appropriateness of the sample *composition* and *size*. It is an important consideration in evaluations of the quality and trustworthiness of much qualitative research [1] and is implicated – particularly for research that is situated within a post-positivist tradition and retains a degree of commitment to realist ontological premises – in appraisals of validity and generalizability [2–5].

Samples in qualitative research tend to be small in order to support the depth of case-oriented analysis that is fundamental to this mode of inquiry [5]. Additionally, qualitative samples are purposive, that is, selected by virtue of their capacity to provide richly-textured information, relevant to the phenomenon under investigation. As a result, purposive sampling [6, 7] – as opposed to probability sampling employed in quantitative research – selects ‘information-rich’ cases [8]. Indeed, recent research demonstrates the greater efficiency of purposive sampling compared to random sampling in qualitative studies [9], supporting related assertions long put forward by qualitative methodologists.

Sample size in qualitative research has been the subject of enduring discussions [4, 10, 11]. Whilst the quantitative research community has established relatively straightforward statistics-based rules to set sample sizes precisely, the intricacies of qualitative sample size determination and assessment arise from the methodological, theoretical, epistemological, and ideological pluralism that characterises qualitative inquiry (for a discussion focused on the discipline of psychology see [12]). This mitigates against clear-cut guidelines, invariably applied. Despite these challenges, various conceptual developments have sought to address this issue, with guidance and principles [4, 10, 11, 13–20], and more recently, an evidence-based approach to sample size determination seeks to ground the discussion empirically [21–35].

Focusing on single-interview-per-participant qualitative designs, the present study aims to further contribute to the dialogue of sample size in qualitative research by offering empirical evidence around justification practices associated with sample size. We next review the existing conceptual and empirical literature on sample size determination.

### Sample size in qualitative research: Conceptual developments and empirical investigations

Qualitative research experts argue that there is no straightforward answer to the question of ‘how many’ and that sample size is contingent on a number of factors relating to epistemological, methodological and practical issues [36]. Sandelowski [4] recommends that qualitative sample sizes are large enough to allow the unfolding of a ‘new and richly textured understanding’ of the phenomenon under study, but small enough so that the ‘deep, case-oriented analysis’ (p. 183) of qualitative data is not precluded. Morse [11] posits that the more useable data are collected from each person, the fewer participants are needed. She invites researchers to take into account parameters, such as the scope of study, the nature of topic (i.e. complexity, accessibility), the quality of data, and the study design. Indeed, the level of structure of questions in qualitative interviewing has been found to influence the richness of data generated [37], and so, requires attention; empirical research shows that open questions, which are asked later on in the interview, tend to produce richer data [37].

Beyond such guidance, specific numerical recommendations have also been proffered, often based on experts’ experience of qualitative research. For example, Green and Thorogood [38] maintain that the experience of most qualitative researchers conducting an interview-based study with a fairly specific research question is that little new information is generated after interviewing 20 people or so belonging to one analytically relevant participant ‘category’ (pp. 102–104). Ritchie et al. [39] suggest that studies employing individual interviews conduct no more than 50 interviews so that researchers are able to manage the complexity of the analytic task. Similarly, Britten [40] notes that large interview studies will often comprise of 50 to 60 people. Experts have also offered numerical guidelines tailored to different theoretical and methodological traditions and specific research approaches, e.g. grounded theory, phenomenology [11, 41]. More recently, a quantitative tool was proposed [42] to support a priori sample size determination based on estimates of the prevalence of themes in the population. Nevertheless, this more formulaic approach raised criticisms relating to assumptions about the conceptual [43] and ontological status of ‘themes’ [44] and the linearity ascribed to the processes of sampling, data collection and data analysis [45].

In terms of principles, Lincoln and Guba [17] proposed that sample size determination be guided by the criterion of *informational redundancy*, that is, sampling can be terminated when no new information is elicited by sampling more units. Following the logic of informational comprehensiveness Malterud et al. [18] introduced the concept of *information power* as a pragmatic guiding principle, suggesting that the more information power the sample provides, the smaller the sample size needs to be, and vice versa.

Undoubtedly, the most widely used principle for determining sample size and evaluating its sufficiency is that of *saturation*. The notion of saturation originates in grounded theory [15] – a qualitative methodological approach explicitly concerned with empirically-derived theory development – and is inextricably linked to theoretical sampling. Theoretical sampling describes an iterative process of data collection, data analysis and theory development whereby data collection is governed by emerging theory rather than predefined characteristics of the population. Grounded theory saturation (often called theoretical saturation) concerns the theoretical categories – as opposed to data – that are being developed and becomes evident when ‘gathering fresh data no longer sparks new theoretical insights, nor reveals new properties of your core theoretical categories’ [46 p. 113]. Saturation in grounded theory, therefore, does not equate to the more common focus on data repetition and moves beyond a singular focus on sample size as the justification of sampling adequacy [46, 47]. Sample size in grounded theory cannot be determined a priori as it is contingent on the evolving theoretical categories.

Saturation – often under the terms of ‘data’ or ‘thematic’ saturation – has diffused into several qualitative communities beyond its origins in grounded theory. Alongside the expansion of its meaning, being variously equated with ‘no new data’, ‘no new themes’, and ‘no new codes’, saturation has emerged as the ‘gold standard’ in qualitative inquiry [2, 26]. Nevertheless, and as Morse [48] asserts, whilst saturation is the most frequently invoked ‘guarantee of qualitative rigor’, ‘it is the one we know least about’ (p. 587). Certainly researchers caution that saturation is less applicable to, or appropriate for, particular types of qualitative research (e.g. conversation analysis, [49]; phenomenological research, [50]) whilst others reject the concept altogether [19, 51].

Methodological studies in this area aim to provide guidance about saturation and develop a practical application of processes that ‘operationalise’ and evidence saturation. Guest, Bunce, and Johnson [26] analysed 60 interviews and found that saturation of themes was reached by the twelfth interview. They noted that their sample was relatively homogeneous, their research aims focused, so studies of more heterogeneous samples and with a broader scope would be likely to need a larger size to achieve saturation. Extending the enquiry to multi-site, cross-cultural research, Hagaman and Wutich [28] showed that sample sizes of 20 to 40 interviews were required to achieve data saturation of meta-themes that cut across research sites. In a theory-driven content analysis, Francis et al. [25] reached data saturation at the 17th interview for all their pre-determined theoretical constructs. The authors further proposed two main principles upon which specification of saturation be based: (a) researchers should a priori specify an *initial analysis sample* (e.g. 10 interviews) which will be used for the first round of analysis and (b) a *stopping criterion*, that is, a number of interviews (e.g. 3) that needs to be further conducted, the analysis of which will not yield any new themes or ideas. For greater transparency, Francis et al. [25] recommend that researchers present cumulative frequency graphs supporting their judgment that saturation was achieved. A comparative method for themes saturation (CoMeTS) has also been suggested [23] whereby the findings of each new interview are compared with those that have already emerged and if it does not yield any new theme, the ‘saturated terrain’ is assumed to have been established. Because the order in which interviews are analysed can influence saturation thresholds depending on the richness of the data, Constantinou et al. [23] recommend reordering and re-analysing interviews to confirm saturation. Hennink, Kaiser and Marconi’s [29] methodological study sheds further light on the problem of specifying and demonstrating saturation. Their analysis of interview data showed that *code saturation* (i.e. the point at which no additional issues are identified) was achieved at 9 interviews, but *meaning saturation* (i.e. the point at which no further dimensions, nuances, or insights of issues are identified) required 16–24 interviews. Although *breadth* can be achieved relatively soon, especially for high-prevalence and concrete codes, *depth* requires additional data, especially for codes of a more conceptual nature.

Critiquing the concept of saturation, Nelson [19] proposes five conceptual depth criteria in grounded theory projects to assess the robustness of the developing theory: (a) theoretical concepts should be supported by a wide range of evidence drawn from the data; (b) be demonstrably part of a network of inter-connected concepts; (c) demonstrate subtlety; (d) resonate with existing literature; and (e) can be successfully submitted to tests of external validity.

Other work has sought to examine practices of sample size reporting and sufficiency assessment across a range of disciplinary fields and research domains, from nutrition [34] and health education [32], to education and the health sciences [22, 27], information systems [30], organisation and workplace studies [33], human computer interaction [21], and accounting studies [24]. Others investigated PhD qualitative studies [31] and grounded theory studies [35]. Incomplete and imprecise sample size reporting is commonly pinpointed by these investigations whilst assessment and justifications of sample size sufficiency are even more sporadic.

Sobal [34] examined the sample size of qualitative studies published in the Journal of Nutrition Education over a period of 30 years. Studies that employed individual interviews (*n* = 30) had an average sample size of 45 individuals and none of these explicitly reported whether their sample size sought and/or attained saturation. A minority of articles discussed how sample-related limitations (with the latter most often concerning the type of sample, rather than the size) limited generalizability. A further systematic analysis [32] of health education research over 20 years demonstrated that interview-based studies averaged 104 participants (range 2 to 720 interviewees). However, 40% did not report the number of participants. An examination of 83 qualitative interview studies in leading information systems journals [30] indicated little defence of sample sizes on the basis of recommendations by qualitative methodologists, prior relevant work, or the criterion of saturation. Rather, sample size seemed to correlate with factors such as the journal of publication or the region of study (US vs Europe vs Asia). These results led the authors to call for more rigor in determining and reporting sample size in qualitative information systems research and to recommend optimal sample size ranges for grounded theory (i.e. 20–30 interviews) and single case (i.e. 15–30 interviews) projects.

Similarly, fewer than 10% of articles in organisation and workplace studies provided a sample size justification relating to existing recommendations by methodologists, prior relevant work, or saturation [33], whilst only 17% of focus groups studies in health-related journals provided an explanation of sample size (i.e. number of focus groups), with saturation being the most frequently invoked argument, followed by published sample size recommendations and practical reasons [22]. The notion of saturation was also invoked by 11 out of the 51 most highly cited studies that Guetterman [27] reviewed in the fields of education and health sciences, of which six were grounded theory studies, four phenomenological and one a narrative inquiry. Finally, analysing 641 interview-based articles in accounting, Dai et al. [24] called for more rigor since a significant minority of studies did not report precise sample size.

Despite increasing attention to rigor in qualitative research (e.g. [52]) and more extensive methodological and analytical disclosures that seek to validate qualitative work [24], sample size reporting and sufficiency assessment remain inconsistent and partial, if not absent, across a range of research domains.

### Objectives of the present study

The present study sought to enrich existing systematic analyses of the customs and practices of sample size reporting and justification by focusing on qualitative research relating to health. Additionally, this study attempted to expand previous empirical investigations by examining how qualitative sample sizes are characterised and discussed in academic narratives. Qualitative health research is an inter-disciplinary field that due to its affiliation with medical sciences, often faces views and positions reflective of a quantitative ethos. Thus qualitative health research constitutes an *emblematic case* that may help to unfold underlying philosophical and methodological differences across the scientific community that are crystallised in considerations of sample size. The present research, therefore, incorporates a comparative element on the basis of three different disciplines engaging with qualitative health research: medicine, psychology, and sociology. We chose to focus our analysis on single-per-participant-interview designs as this not only presents a popular and widespread methodological choice in qualitative health research, but also as the method where consideration of sample size – defined as the number of interviewees – is particularly salient.

## Methods

### Study design

A structured search for articles reporting cross-sectional, interview-based qualitative studies was carried out and eligible reports were systematically reviewed and analysed employing both quantitative and qualitative analytic techniques.

We selected journals which (a) follow a peer review process, (b) are considered high quality and influential in their field as reflected in journal metrics, and (c) are receptive to, and publish, qualitative research (Additional File 1 presents the journals’ editorial positions in relation to qualitative research and sample considerations where available). Three health-related journals were chosen, each representing a different disciplinary field; the *British Medical Journal* (BMJ) representing medicine, the *British Journal of Health Psychology* (BJHP) representing psychology, and the *Sociology of Health & Illness* (SHI) representing sociology.

### Search strategy to identify studies

Employing the search function of each individual journal, we used the terms ‘interview*’ AND ‘qualitative’ and limited the results to articles published between 1 January 2003 and 22 September 2017 (i.e. a 15-year review period).

### Eligibility criteria

**Fig. 1:**
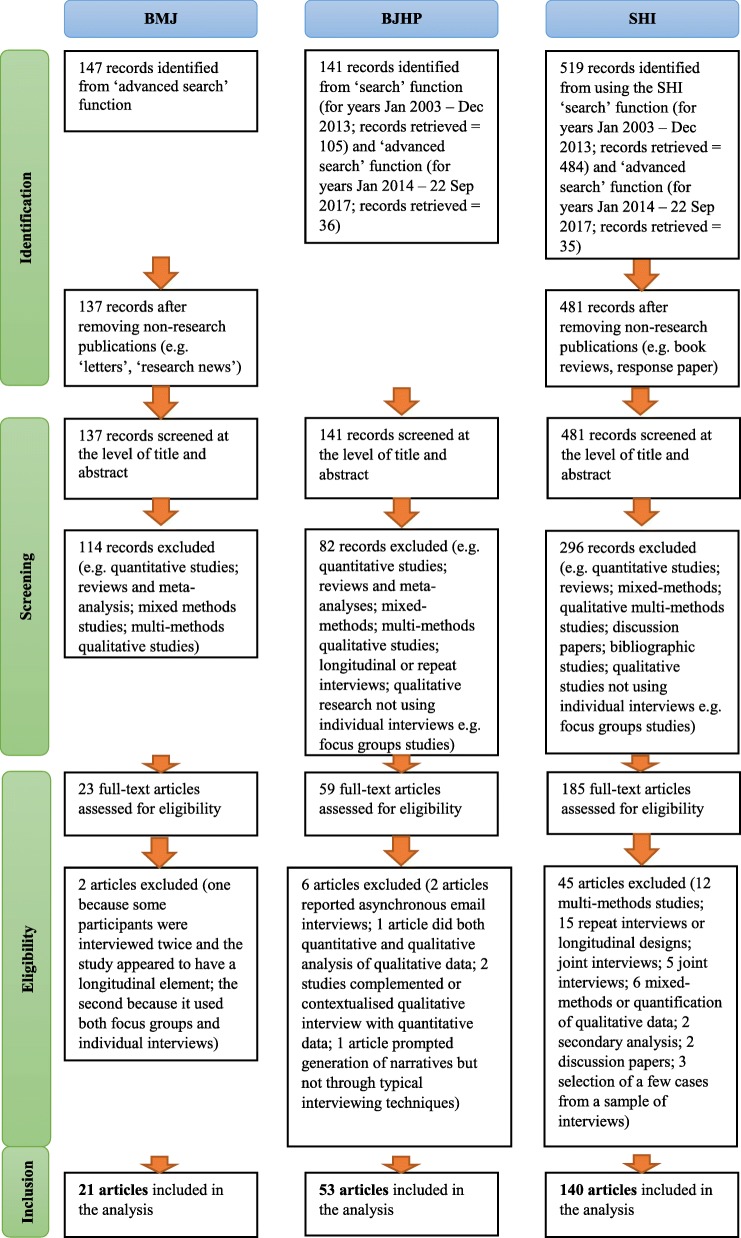
PRISMA flow diagram

To be eligible for inclusion in the review, the article had to report a cross-sectional study design. Longitudinal studies were thus excluded whilst studies conducted within a broader research programme (e.g. interview studies nested in a trial, as part of a broader ethnography, as part of a longitudinal research) were included if they reported only single-time qualitative interviews. The method of data collection had to be individual, synchronous qualitative interviews (i.e. group interviews, structured interviews and e-mail interviews over a period of time were excluded), and the data had to be analysed qualitatively (i.e. studies that quantified their qualitative data were excluded). Mixed method studies and articles reporting more than one qualitative method of data collection (e.g. individual interviews and focus groups) were excluded. Figure 1, a PRISMA flow diagram [53], shows the number of: articles obtained from the searches and screened; papers assessed for eligibility; and articles included in the review (Additional File 2 provides the full list of articles included in the review and their unique identifying code – e.g. BMJ01, BJHP02, SHI03). One review author (KV) assessed the eligibility of all papers identified from the searches. When in doubt, discussions about retaining or excluding articles were held between KV and JB in regular meetings, and decisions were jointly made.

### Data extraction and analysis

A data extraction form was developed (see Additional File 3) recording three areas of information: (a) information about the article (e.g. authors, title, journal, year of publication etc.); (b) information about the aims of the study, the sample size and any justification for this, the participant characteristics, the sampling technique and any sample-related observations or comments made by the authors; and (c) information about the method or technique(s) of data analysis, the number of researchers involved in the analysis, the potential use of software, and any discussion around epistemological considerations. The Abstract, Methods and Discussion (and/or Conclusion) sections of each article were examined by one author (KV) who extracted all the relevant information. This was directly copied from the articles and, when appropriate, comments, notes and initial thoughts were written down.

To examine the kinds of sample size justifications provided by articles, an inductive content analysis [54] was initially conducted. On the basis of this analysis, the categories that expressed qualitatively different sample size justifications were developed.

We also extracted or coded quantitative data regarding the following aspects:

- Journal and year of publication
- Number of interviews
- Number of participants
- Presence of sample size justification(s) (Yes/No)
- Presence of a particular sample size justification category (Yes/No), and
- Number of sample size justifications provided

Descriptive and inferential statistical analyses were used to explore these data.

A thematic analysis [55] was then performed on all scientific narratives that discussed or commented on the sample size of the study. These narratives were evident both in papers that justified their sample size and those that did not. To identify these narratives, in addition to the methods sections, the discussion sections of the reviewed articles were also examined and relevant data were extracted and analysed.

## Results

**Fig. 2:**
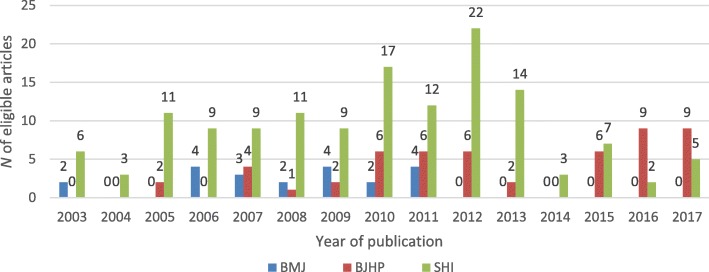
Number of eligible articles published each year per journal^2^

In total, 214 articles – 21 in the BMJ, 53 in the BJHP and 140 in the SHI – were eligible for inclusion in the review. Table 1 provides basic information about the sample sizes – measured in number of interviews – of the studies reviewed across the three journals. Figure 2 depicts the number of eligible articles published each year per journal.

**Table 1:** Descriptive statistics of the sample sizes of eligible articles across the three journals

Sample size of studies	BMJ (*n* = 21)	BJHP (*n* = 53)	SHI (*n* = 140)
*Mean (SD)* number of interviews	44.5 (29.3)	18.1 (10.4)	37.4 (28)
*Min* number of interviews	19	6	7
*Max* number of interviews	128	55	197
*Median*	31	15	30.5

Pairwise comparisons following a significant Kruskal-Wallis^1^ test indicated that the studies published in the BJHP had significantly (*p* < .001) smaller samples sizes than those published either in the BMJ or the SHI. Sample sizes of BMJ and SHI articles did not differ significantly from each other.

### Sample size justifications: Results from the quantitative and qualitative content analysis

Ten (47.6%) of the 21 BMJ studies, 26 (49.1%) of the 53 BJHP papers and 24 (17.1%) of the 140 SHI articles provided some sort of sample size justification. As shown in Table 2, the majority of articles which justified their sample size provided one justification (70% of articles); fourteen studies (25%) provided two distinct justifications; one study (1.7%) gave three justifications and two studies (3.3%) expressed four distinct justifications.

**Table 2:** Number and percentage of ‘justifying’ articles and number of justifications stated by ‘justifying’ articles

How many justifications were provided by the ‘justifying’ articles?	BMJ	BJHP	SHI	Total
One justification	6	17	19	42 (70%)
Two justifications	2	8	5	15 (25%)
Three justifications	1	0	0	1 (1.7%)
Four justifications	1	1	0	2 (3.3%)
Total *N* of ‘justifying’ articles	10	26	24	60
(out of eligible articles)	(21)	(53)	(140)	(214)
% of ‘justifying’ articles	47.6	49.1	17.1	28

There was no association between the number of interviews (i.e. sample size) conducted and the provision of a justification (rpb = .054, *p* = .433). Within journals, Mann-Whitney tests indicated that sample sizes of ‘justifying’ and ‘non-justifying’ articles in the BMJ and SHI did not differ significantly from each other. In the BJHP, ‘justifying’ articles (*Mean rank* = 31.3) had significantly larger sample sizes than ‘non-justifying’ studies (*Mean rank* = 22.7; U = 237.000, *p* < .05).

There was a significant association between the journal a paper was published in and the provision of a justification (χ^2^ (2) = 23.83, *p* < .001). BJHP studies provided a sample size justification significantly more often than would be expected (*z* = 2.9); SHI studies significantly less often (*z* = − 2.4). If an article was published in the BJHP, the odds of providing a justification were 4.8 times higher than if published in the SHI. Similarly if published in the BMJ, the odds of a study justifying its sample size were 4.5 times higher than in the SHI.

The qualitative content analysis of the scientific narratives identified eleven different sample size justifications. These are described below and illustrated with excerpts from relevant articles. By way of a summary, the frequency with which these were deployed across the three journals is indicated in Table 3.

**Table 3:** Commonality, type and counts of sample size justifications across journals

Commonality of justifications across journals	Qualitatively different justifications	BMJ	BJHP	SHI	Total
Justifications shared by all 3 journals	1. Saturation	7	20	19	46
	2. Pragmatic considerations	1	4	3	8
Justifications shared by 2 journals	3. Qualities of the analysis	1	6	0	7
	4. Meet sampling requirements	2	0	4	6
	5. Sample size guidelines	0	5	1	6
	6. In line with existing research	2	1	0	3
	7. Richness and volume of data	1	0	1	2
Justifications found in 1 journal only	8. Meet research design requirements	2	0	0	2
	9. Researchers’ previous experience	1	0	0	1
	10. Nature of study	0	1	0	1
	11. Further sampling to check findings consistency	0	0	1	1
	*Total*	*17*	*37*	*29*	*83*

### Saturation

Saturation was the most commonly invoked principle (55.4% of all justifications) deployed by studies across all three journals to justify the sufficiency of their sample size. In the BMJ, two studies claimed that they achieved *data saturation* (BMJ17; BMJ18) and one article referred descriptively to achieving saturation without explicitly using the term (BMJ13). Interestingly, BMJ13 included data in the analysis beyond the point of saturation in search of ‘unusual/deviant observations’ and with a view to establishing findings consistency.

*Thirty three women were approached to take part in the interview study. Twenty seven agreed and 21 (aged 21–64, median 40) were interviewed before data saturation was reached (one tape failure meant that 20 interviews were available for analysis).* (BMJ17).

*No new topics were identified following analysis of approximately two thirds of the interviews; however, all interviews were coded in order to develop a better understanding of how characteristic the views and reported behaviours were, and also to collect further examples of unusual/deviant observations.* (BMJ13).

Two articles reported pre-determining their sample size with a view to achieving data saturation (BMJ08 – see extract in section *In line with existing research*; BMJ15 – see extract in section *Pragmatic considerations*) without further specifying if this was achieved. One paper claimed *theoretical saturation* (BMJ06) conceived as being when “no further recurring themes emerging from the analysis” whilst another study argued that although the analytic categories were highly saturated, it was not possible to determine whether theoretical saturation had been achieved (BMJ04). One article (BMJ18) cited a reference to support its position on saturation.

In the BJHP, six articles claimed that they achieved *data saturation* (BJHP21; BJHP32; BJHP39; BJHP48; BJHP49; BJHP52) and one article stated that, given their sample size and the guidelines for achieving data saturation, it anticipated that saturation would be attained (BJHP50).

*Recruitment continued until data saturation was reached, defined as the point at which no new themes emerged.* (BJHP48).

*It has previously been recommended that qualitative studies require a minimum sample size of at least 12 to reach data saturation (Clarke & Braun, 2013; Fugard & Potts, 2014; Guest, Bunce, & Johnson, 2006) Therefore, a sample of 13 was deemed sufficient for the qualitative analysis and scale of this study.* (BJHP50).

Two studies argued that they achieved *thematic saturation* (BJHP28 – see extract in section *Sample size guidelines*; BJHP31) and one (BJHP30) article, explicitly concerned with theory development and deploying theoretical sampling, claimed both theoretical and data saturation.

*The final sample size was determined by thematic saturation, the point at which new data appears to no longer contribute to the findings due to repetition of themes and comments by participants (Morse, 1995). At this point, data generation was terminated.* (BJHP31).

Five studies argued that they achieved (BJHP05; BJHP33; BJHP40; BJHP13 – see extract in section *Pragmatic considerations*) or anticipated (BJHP46) saturation without any further specification of the term. BJHP17 referred descriptively to a state of achieved saturation without specifically using the term. *Saturation of coding*, but not saturation of themes, was claimed to have been reached by one article (BJHP18). Two articles explicitly stated that they did not achieve saturation; instead claiming a level of *theme completeness* (BJHP27) or that themes being replicated (BJHP53) were arguments for sufficiency of their sample size.

*Furthermore, data collection ceased on pragmatic grounds rather than at the point when saturation point was reached. Despite this, although nuances within sub-themes were still emerging towards the end of data analysis, the themes themselves were being replicated indicating a level of completeness.* (BJHP27).

Finally, one article criticised and explicitly renounced the notion of data saturation claiming that, on the contrary, the criterion of *theoretical sufficiency* determined its sample size (BJHP16).

*According to the original Grounded Theory texts, data collection should continue until there are no new discoveries (*i.e.*, ‘data saturation’; Glaser & Strauss, 1967). However, recent revisions of this process have discussed how it is rare that data collection is an exhaustive process and researchers should rely on how well their data are able to create a sufficient theoretical account or ‘theoretical sufficiency’ (Dey, 1999). For this study, it was decided that theoretical sufficiency would guide recruitment, rather than looking for data saturation.* (BJHP16).

Ten out of the 20 BJHP articles that employed the argument of saturation used one or more citations relating to this principle.

In the SHI, one article (SHI01) claimed that it achieved *category saturation* based on authors’ judgment.

*This number was not fixed in advance, but was guided by the sampling strategy and the judgement, based on the analysis of the data, of the point at which ‘category saturation’ was achieved.* (SHI01).

Three articles described a state of achieved saturation without using the term or specifying what sort of saturation they had achieved (i.e. data, theoretical, thematic saturation) (SHI04; SHI13; SHI30) whilst another four articles explicitly stated that they achieved saturation (SHI100; SHI125; SHI136; SHI137). Two papers stated that they achieved *data saturation* (SHI73 – see extract in section *Sample size guidelines*; SHI113), two claimed *theoretical saturation* (SHI78; SHI115) and two referred to achieving *thematic saturation* (SHI87; SHI139) or to *saturated themes* (SHI29; SHI50).

*Recruitment and analysis ceased once theoretical saturation was reached in the categories described below (Lincoln and Guba 1985).* (SHI115).

*The respondents’ quotes drawn on below were chosen as representative, and illustrate saturated themes.* (SHI50).

One article stated that thematic saturation was anticipated with its sample size (SHI94). Briefly referring to the difficulty in pinpointing achievement of theoretical saturation, SHI32 (see extract in section *Richness and volume of data*) defended the sufficiency of its sample size on the basis of “the high degree of consensus [that] had begun to emerge among those interviewed”, suggesting that information from interviews was being replicated. Finally, SHI112 (see extract in section *Further sampling to check findings consistency*) argued that it achieved *saturation of discursive patterns*. Seven of the 19 SHI articles cited references to support their position on saturation (see Additional File 4 for the full list of citations used by articles to support their position on saturation across the three journals).

Overall, it is clear that the concept of saturation encompassed a wide range of variants expressed in terms such as saturation, data saturation, thematic saturation, theoretical saturation, category saturation, saturation of coding, saturation of discursive themes, theme completeness. It is noteworthy, however, that although these various claims were sometimes supported with reference to the literature, they were not evidenced in relation to the study at hand.

### Pragmatic considerations

The determination of sample size on the basis of pragmatic considerations was the second most frequently invoked argument (9.6% of all justifications) appearing in all three journals. In the BMJ, one article (BMJ15) appealed to pragmatic reasons, relating to time constraints and the difficulty to access certain study populations, to justify the determination of its sample size.

*On the basis of the researchers’ previous experience and the literature,* *[30, 31]* *we estimated that recruitment of 15–20 patients at each site would achieve data saturation when data from each site were analysed separately. We set a target of seven to 10 caregivers per site because of time constraints and the anticipated difficulty of accessing caregivers at some home based care services. This gave a target sample of 75–100 patients and 35–50 caregivers overall.* (BMJ15).

In the BJHP, four articles mentioned pragmatic considerations relating to time or financial constraints (BJHP27 – see extract in section *Saturation*; BJHP53), the participant response rate (BJHP13), and the fixed (and thus limited) size of the participant pool from which interviewees were sampled (BJHP18).

*We had aimed to continue interviewing until we had reached saturation, a point whereby further data collection would yield no further themes. In practice, the number of individuals volunteering to participate dictated when recruitment into the study ceased (15 young people, 15 parents). Nonetheless, by the last few interviews, significant repetition of concepts was occurring, suggesting ample sampling.* (BJHP13).

Finally, three SHI articles explained their sample size with reference to practical aspects: time constraints and project manageability (SHI56), limited availability of respondents and project resources (SHI131), and time constraints (SHI113).

*The size of the sample was largely determined by the availability of respondents and resources to complete the study. Its composition reflected, as far as practicable, our interest in how contextual factors (for example, gender relations and ethnicity) mediated the illness experience.* (SHI131).

### Qualities of the analysis

This sample size justification (8.4% of all justifications) was mainly employed by BJHP articles and referred to an intensive, idiographic and/or latently focused analysis, i.e. that moved beyond description. More specifically, six articles defended their sample size on the basis of an intensive analysis of transcripts and/or the idiographic focus of the study/analysis. Four of these papers (BJHP02; BJHP19; BJHP24; BJHP47) adopted an Interpretative Phenomenological Analysis (IPA) approach.

*The current study employed a sample of 10 in keeping with the aim of exploring each participant’s account (Smith* et al.*, 1999).* (BJHP19).

BJHP47 explicitly renounced the notion of saturation within an IPA approach. The other two BJHP articles conducted thematic analysis (BJHP34; BJHP38). The level of analysis – i.e. latent as opposed to a more superficial descriptive analysis – was also invoked as a justification by BJHP38 alongside the argument of an intensive analysis of individual transcripts

*The resulting sample size was at the lower end of the range of sample sizes employed in thematic analysis (Braun & Clarke, 2013). This was in order to enable significant reflection, dialogue, and time on each transcript and was in line with the more latent level of analysis employed, to identify underlying ideas, rather than a more superficial descriptive analysis (Braun & Clarke, 2006).* (BJHP38).

Finally, one BMJ paper (BMJ21) defended its sample size with reference to the complexity of the analytic task.

*We stopped recruitment when we reached 30–35 interviews, owing to the depth and duration of interviews, richness of data, and complexity of the analytical task.* (BMJ21).

### Meet sampling requirements

Meeting sampling requirements (7.2% of all justifications) was another argument employed by two BMJ and four SHI articles to explain their sample size. Achieving maximum variation sampling in terms of specific interviewee characteristics determined and explained the sample size of two BMJ studies (BMJ02; BMJ16 – see extract in section *Meet research design requirements*).

*Recruitment continued until sampling frame requirements were met for diversity in age, sex, ethnicity, frequency of attendance, and health status.* (BMJ02).

Regarding the SHI articles, two papers explained their numbers on the basis of their sampling strategy (SHI01- see extract in section *Saturation*; SHI23) whilst sampling requirements that would help attain sample heterogeneity in terms of a particular characteristic of interest was cited by one paper (SHI127).

*The combination of matching the recruitment sites for the quantitative research and the additional purposive criteria led to 104 phase 2 interviews (Internet (OLC): 21; Internet (FTF): 20); Gyms (FTF): 23; HIV testing (FTF): 20; HIV treatment (FTF): 20.)* (SHI23).

*Of the fifty interviews conducted, thirty were translated from Spanish into English. These thirty, from which we draw our findings, were chosen for translation based on heterogeneity in depressive symptomology and educational attainment.* (SHI127).

Finally, the pre-determination of sample size on the basis of sampling requirements was stated by one article though this was not used to justify the number of interviews (SHI10).

### Sample size guidelines

Five BJHP articles (BJHP28; BJHP38 – see extract in section *Qualities of the analysis*; BJHP46; BJHP47; BJHP50 – see extract in section *Saturation*) and one SHI paper (SHI73) relied on citing existing sample size guidelines or norms within research traditions to determine and subsequently defend their sample size (7.2% of all justifications).

*Sample size guidelines suggested a range between 20 and 30 interviews to be adequate (Creswell, 1998). Interviewer and note taker agreed that thematic saturation, the point at which no new concepts emerge from subsequent interviews (Patton, 2002), was achieved following completion of 20 interviews.* (BJHP28).

*Interviewing continued until we deemed data saturation to have been reached (the point at which no new themes were emerging). Researchers have proposed 30 as an approximate or working number of interviews at which one could expect to be reaching theoretical saturation when using a semi-structured interview approach (Morse 2000), although this can vary depending on the heterogeneity of respondents interviewed and complexity of the issues explored.* (SHI73).

### In line with existing research

Sample sizes of published literature in the area of the subject matter under investigation (3.5% of all justifications) were used by 2 BMJ articles as guidance and a precedent for determining and defending their own sample size (BMJ08; BMJ15 – see extract in section *Pragmatic considerations*).

*We drew participants from a list of prisoners who were scheduled for release each week, sampling them until we reached the target of 35 cases, with a view to achieving data saturation within the scope of the study and sufficient follow-up interviews and in line with recent studies* *[8–10].* (BMJ08).

Similarly, BJHP38 (see extract in section *Qualities of the analysis*) claimed that its sample size was within the range of sample sizes of published studies that use its analytic approach.

### Richness and volume of data

BMJ21 (see extract in section *Qualities of the analysis*) and SHI32 referred to the richness, detailed nature, and volume of data collected (2.3% of all justifications) to justify the sufficiency of their sample size.

*Although there were more potential interviewees from those contacted by postcode selection, it was decided to stop recruitment after the 10th interview and focus on analysis of this sample. The material collected was considerable and, given the focused nature of the study, extremely detailed. Moreover, a high degree of consensus had begun to emerge among those interviewed, and while it is always difficult to judge at what point ‘theoretical saturation’ has been reached, or how many interviews would be required to uncover exception(s), it was felt the number was sufficient to satisfy the aims of this small in-depth investigation (Strauss and Corbin 1990).* (SHI32).

### Meet research design requirements

Determination of sample size so that it is in line with, and serves the requirements of, the research design (2.3% of all justifications) that the study adopted was another justification used by 2 BMJ papers (BMJ16; BMJ08 – see extract in section *In line with existing research*).

*We aimed for diverse, maximum variation samples* *[20]* *totalling 80 respondents from different social backgrounds and ethnic groups and those bereaved due to different types of suicide and traumatic death. We could have interviewed a smaller sample at different points in time (a qualitative longitudinal study) but chose instead to seek a broad range of experiences by interviewing those bereaved many years ago and others bereaved more recently; those bereaved in different circumstances and with different relations to the deceased; and people who lived in different parts of the UK; with different support systems and coroners’ procedures (see* *Tables 1 and 2 for more details).* (BMJ16).

### Researchers’ previous experience

The researchers’ previous experience (possibly referring to experience with qualitative research) was invoked by BMJ15 (see extract in section *Pragmatic considerations*) as a justification for the determination of sample size.

### Nature of study

One BJHP paper argued that the sample size was appropriate for the exploratory nature of the study (BJHP38).

*A sample of eight participants was deemed appropriate because of the exploratory nature of this research and the focus on identifying underlying ideas about the topic.* (BJHP38).

### Further sampling to check findings consistency

Finally, SHI112 argued that once it had achieved saturation of discursive patterns, further sampling was decided and conducted to check for consistency of the findings.

*Within each of the age-stratified groups, interviews were randomly sampled until saturation of discursive patterns was achieved. This resulted in a sample of 67 interviews. Once this sample had been analysed, one further interview from each age-stratified group was randomly chosen to check for consistency of the findings. Using this approach it was possible to more carefully explore children’s discourse about the ‘I’, agency, relationality and power in the thematic areas, revealing the subtle discursive variations described in this article.* (SHI112).

### Thematic analysis of passages discussing sample size

This analysis resulted in two overarching thematic areas; the first concerned the variation in the characterisation of sample size sufficiency, and the second related to the perceived threats deriving from sample size insufficiency.

### Characterisations of sample size sufficiency

The analysis showed that there were three main characterisations of the sample size in the articles that provided relevant comments and discussion: (a) the vast majority of these qualitative studies (*n* = 42) considered their sample size as ‘small’ and this was seen and discussed as a limitation; only two articles viewed their small sample size as desirable and appropriate (b) a minority of articles (*n* = 4) proclaimed that their achieved sample size was ‘sufficient’; and (c) finally, a small group of studies (*n* = 5) characterised their sample size as ‘large’. Whilst achieving a ‘large’ sample size was sometimes viewed positively because it led to richer results, there were also occasions when a large sample size was problematic rather than desirable.

#### ‘Small’ but why and for whom?

A number of articles which characterised their sample size as ‘small’ did so against an implicit or explicit quantitative framework of reference. Interestingly, three studies that claimed to have achieved data saturation or ‘theoretical sufficiency’ with their sample size, discussed or noted as a limitation in their discussion their ‘small’ sample size, raising the question of why, or for whom, the sample size was considered small given that the qualitative criterion of saturation had been satisfied.

*The current study has a number of limitations. The sample size was small (n = 11) and, however, large enough for no new themes to emerge.* (BJHP39).

*The study has two principal limitations. The first of these relates to the small number of respondents who took part in the study.* (SHI73).

Other articles appeared to accept and acknowledge that their sample was flawed because of its small size (as well as other compositional ‘deficits’ e.g. non-representativeness, biases, self-selection) or anticipated that they might be criticized for their small sample size. It seemed that the imagined audience – perhaps reviewer or reader – was one inclined to hold the tenets of quantitative research, and certainly one to whom it was important to indicate the recognition that small samples were likely to be problematic. That one’s sample might be thought small was often construed as a limitation couched in a discourse of regret or apology.

Very occasionally, the articulation of the small size as a limitation was explicitly aligned against an espoused positivist framework and quantitative research.

*This study has some limitations. Firstly, the 100 incidents sample represents a small number of the total number of serious incidents that occurs every year.*^*26*^ *We sent out a nationwide invitation and do not know why more people did not volunteer for the study. Our lack of epidemiological knowledge about healthcare incidents, however, means that determining an appropriate sample size continues to be difficult.* (BMJ20).

Indicative of an apparent oscillation of qualitative researchers between the different requirements and protocols demarcating the quantitative and qualitative worlds, there were a few instances of articles which briefly recognised their ‘small’ sample size as a limitation, but then defended their study on more qualitative grounds, such as their ability and success at capturing the complexity of experience and delving into the idiographic, and at generating particularly rich data.

*This research, while limited in size, has sought to capture some of the complexity attached to men’s attitudes and experiences concerning incomes and material circumstances.* (SHI35).

*Our numbers are small because negotiating access to social networks was slow and labour intensive, but our methods generated exceptionally rich data.* (BMJ21).

*This study could be criticised for using a small and unrepresentative sample. Given that older adults have been ignored in the research concerning suntanning, fair-skinned older adults are the most likely to experience skin cancer, and women privilege appearance over health when it comes to sunbathing practices, our study offers depth and richness of data in a demographic group much in need of research attention.* (SHI57).

#### ‘Good enough’ sample sizes

Only four articles expressed some degree of confidence that their achieved sample size was sufficient. For example, SHI139, in line with the justification of thematic saturation that it offered, expressed trust in its sample size sufficiency despite the poor response rate. Similarly, BJHP04, which did not provide a sample size justification, argued that it targeted a larger sample size in order to eventually recruit a sufficient number of interviewees, due to anticipated low response rate.

*Twenty-three people with type I diabetes from the target population of 133 (*i.e. *17.3%) consented to participate but four did not then respond to further contacts (total N = 19). The relatively low response rate was anticipated, due to the busy life-styles of young people in the age range, the geographical constraints, and the time required to participate in a semi-structured interview, so a larger target sample allowed a sufficient number of participants to be recruited.* (BJHP04).

Two other articles (BJHP35; SHI32) linked the claimed sufficiency to the scope (i.e. ‘small, in-depth investigation’), aims and nature (i.e. ‘exploratory’) of their studies, thus anchoring their numbers to the particular context of their research. Nevertheless, claims of sample size sufficiency were sometimes undermined when they were juxtaposed with an acknowledgement that a larger sample size would be more scientifically productive.

*Although our sample size was sufficient for this exploratory study, a more diverse sample including participants with lower socioeconomic status and more ethnic variation would be informative. A larger sample could also ensure inclusion of a more representative range of apps operating on a wider range of platforms.* (BJHP35).

#### ‘Large’ sample sizes - Promise or peril?

Three articles (BMJ13; BJHP05; BJHP48) which all provided the justification of saturation, characterised their sample size as ‘large’ and narrated this oversufficiency in positive terms as it allowed richer data and findings and enhanced the potential for generalisation. The type of generalisation aspired to (BJHP48) was not further specified however.

*This study used rich data provided by a relatively large sample of expert informants on an important but under-researched topic.* (BMJ13).

*Qualitative research provides a unique opportunity to understand a clinical problem from the patient’s perspective. This study had a large diverse sample, recruited through a range of locations and used in-depth interviews which enhance the richness and generalizability of the results.* (BJHP48).

And whilst a ‘large’ sample size was endorsed and valued by some qualitative researchers, within the psychological tradition of IPA, a ‘large’ sample size was counter-normative and therefore needed to be justified. Four BJHP studies, all adopting IPA, expressed the appropriateness or desirability of ‘small’ sample sizes (BJHP41; BJHP45) or hastened to explain why they included a larger than typical sample size (BJHP32; BJHP47). For example, BJHP32 below provides a rationale for how an IPA study can accommodate a large sample size and how this was indeed suitable for the purposes of the particular research. To strengthen the explanation for choosing a non-normative sample size, previous IPA research citing a similar sample size approach is used as a precedent.

*Small scale IPA studies allow in-depth analysis which would not be possible with larger samples (Smith* et al.*, 2009).* (BJHP41).

*Although IPA generally involves intense scrutiny of a small number of transcripts, it was decided to recruit a larger diverse sample as this is the first qualitative study of this population in the United Kingdom (as far as we know) and we wanted to gain an overview. Indeed, Smith, Flowers, and Larkin (2009) agree that IPA is suitable for larger groups. However, the emphasis changes from an in-depth individualistic analysis to one in which common themes from shared experiences of a group of people can be elicited and used to understand the network of relationships between themes that emerge from the interviews. This large-scale format of IPA has been used by other researchers in the field of false-positive research. Baillie, Smith, Hewison, and Mason (2000) conducted an IPA study, with 24 participants, of ultrasound screening for chromosomal abnormality; they found that this larger number of participants enabled them to produce a more refined and cohesive account.* (BJHP32).

The IPA articles found in the BJHP were the only instances where a ‘small’ sample size was advocated and a ‘large’ sample size problematized and defended. These IPA studies illustrate that the characterisation of sample size sufficiency can be a function of researchers’ theoretical and epistemological commitments rather than the result of an ‘objective’ sample size assessment.

### Threats from sample size insufficiency

As shown above, the majority of articles that commented on their sample size, simultaneously characterized it as small and problematic. On those occasions that authors did not simply cite their ‘small’ sample size as a study limitation but rather continued and provided an account of how and why a small sample size was problematic, two important scientific qualities of the research seemed to be threatened: the generalizability and validity of results.

#### Generalizability

Those who characterised their sample as ‘small’ connected this to the limited potential for generalization of the results. Other features related to the sample – often some kind of compositional particularity – were also linked to limited potential for generalisation. Though not always explicitly articulated to what form of generalisation the articles referred to (see BJHP09), generalisation was mostly conceived in nomothetic terms, that is, it concerned the potential to draw inferences from the sample to the broader study population (‘representational generalisation’ – see BJHP31) and less often to other populations or cultures.

*It must be noted that samples are small and whilst in both groups the majority of those women eligible participated, generalizability cannot be assumed.* (BJHP09).

*The study’s limitations should be acknowledged: Data are presented from interviews with a relatively small group of participants, and thus, the views are not necessarily generalizable to all patients and clinicians. In particular, patients were only recruited from secondary care services where COFP diagnoses are typically confirmed. The sample therefore is unlikely to represent the full spectrum of patients, particularly those who are not referred to, or who have been discharged from dental services.* (BJHP31).

Without explicitly using the term generalisation, two SHI articles noted how their ‘small’ sample size imposed limits on ‘the extent that we can extrapolate from these participants’ accounts’ (SHI114) or to the possibility ‘to draw far-reaching conclusions from the results’ (SHI124).

Interestingly, only a minority of articles alluded to, or invoked, a type of generalisation that is aligned with qualitative research, that is, idiographic generalisation (i.e. generalisation that can be made *from and about cases* [5]). These articles, all published in the discipline of sociology, defended their findings in terms of the possibility of drawing logical and conceptual inferences to other contexts and of generating understanding that has the potential to advance knowledge, despite their ‘small’ size. One article (SHI139) clearly contrasted nomothetic (statistical) generalisation to idiographic generalisation, arguing that the lack of statistical generalizability does not nullify the ability of qualitative research to still be relevant beyond the sample studied.

*Further, these data do not need to be statistically generalisable for us to draw inferences that may advance medicalisation analyses (Charmaz 2014). These data may be seen as an opportunity to generate further hypotheses and are a unique application of the medicalisation framework.* (SHI139).

*Although a small-scale qualitative study related to school counselling, this analysis can be usefully regarded as a case study of the successful utilisation of mental health-related resources by adolescents. As many of the issues explored are of relevance to mental health stigma more generally, it may also provide insights into adult engagement in services. It shows how a sociological analysis, which uses positioning theory to examine how people negotiate, partially accept and simultaneously resist stigmatisation in relation to mental health concerns, can contribute to an elucidation of the social processes and narrative constructions which may maintain as well as bridge the mental health service gap.* (SHI103).

Only one article (SHI30) used the term *transferability* to argue for the potential of wider relevance of the results which was thought to be more the product of the composition of the sample (i.e. diverse sample), rather than the sample size.

#### Validity

The second major concern that arose from a ‘small’ sample size pertained to the internal validity of findings (i.e. here the term is used to denote the ‘truth’ or credibility of research findings). Authors expressed uncertainty about the degree of confidence in particular aspects or patterns of their results, primarily those that concerned some form of differentiation on the basis of relevant participant characteristics.

*The information source preferred seemed to vary according to parents’ education; however, the sample size is too small to draw conclusions about such patterns.* (SHI80).

*Although our numbers were too small to demonstrate gender differences with any certainty, it does seem that the biomedical and erotic scripts may be more common in the accounts of men and the relational script more common in the accounts of women.* (SHI81).

In other instances, articles expressed uncertainty about whether their results accounted for the full spectrum and variation of the phenomenon under investigation. In other words, a ‘small’ sample size (alongside compositional ‘deficits’ such as a not statistically representative sample) was seen to threaten the ‘content validity’ of the results which in turn led to constructions of the study conclusions as tentative.

*Data collection ceased on pragmatic grounds rather than when no new information appeared to be obtained (*i.e.*, saturation point). As such, care should be taken not to overstate the findings. Whilst the themes from the initial interviews seemed to be replicated in the later interviews, further interviews may have identified additional themes or provided more nuanced explanations.* (BJHP53).

*…it should be acknowledged that this study was based on a small sample of self-selected couples in enduring marriages who were not broadly representative of the population. Thus, participants may not be representative of couples that experience postnatal PTSD. It is therefore unlikely that all the key themes have been identified and explored. For example, couples who were excluded from the study because the male partner declined to participate may have been experiencing greater interpersonal difficulties.* (BJHP03).

In other instances, articles attempted to preserve a degree of credibility of their results, despite the recognition that the sample size was ‘small’. Clarity and sharpness of emerging themes and alignment with previous relevant work were the arguments employed to warrant the validity of the results.

*This study focused on British Chinese carers of patients with affective disorders, using a qualitative methodology to synthesise the sociocultural representations of illness within this community. Despite the small sample size, clear themes emerged from the narratives that were sufficient for this exploratory investigation.* (SHI98).

## Discussion

The present study sought to examine how qualitative sample sizes in health-related research are characterised and justified. In line with previous studies [22, 30, 33, 34] the findings demonstrate that reporting of sample size sufficiency is limited; just over 50% of articles in the BMJ and BJHP and 82% in the SHI did not provide any sample size justification. Providing a sample size justification was not related to the number of interviews conducted, but it was associated with the journal that the article was published in, indicating the influence of disciplinary or publishing norms, also reported in prior research [30]. This lack of transparency about sample size sufficiency is problematic given that most qualitative researchers would agree that it is an important marker of quality [56, 57]. Moreover, and with the rise of qualitative research in social sciences, efforts to synthesise existing evidence and assess its quality are obstructed by poor reporting [58, 59].

When authors justified their sample size, our findings indicate that sufficiency was mostly appraised with reference to features that were intrinsic to the study, in agreement with general advice on sample size determination [4, 11, 36]. The principle of saturation was the most commonly invoked argument [22] accounting for 55% of all justifications. A wide range of variants of saturation was evident corroborating the proliferation of the meaning of the term [49] and reflecting different underlying conceptualisations or models of saturation [20]. Nevertheless, claims of saturation were never substantiated in relation to procedures conducted in the study itself, endorsing similar observations in the literature [25, 30, 47]. Claims of saturation were sometimes supported with citations of other literature, suggesting a removal of the concept away from the characteristics of the study at hand. Pragmatic considerations, such as resource constraints or participant response rate and availability, was the second most frequently used argument accounting for approximately 10% of justifications and another 23% of justifications also represented intrinsic-to-the-study characteristics (i.e. qualities of the analysis, meeting sampling or research design requirements, richness and volume of the data obtained, nature of study, further sampling to check findings consistency).

Only, 12% of mentions of sample size justification pertained to arguments that were external to the study at hand, in the form of existing sample size guidelines and prior research that sets precedents. Whilst community norms and prior research can establish useful rules of thumb for estimating sample sizes [60] – and reveal what sizes are more likely to be acceptable within research communities – researchers should avoid adopting these norms uncritically, especially when such guidelines [e.g. 30, 35], might be based on research that does not provide adequate evidence of sample size sufficiency. Similarly, whilst methodological research that seeks to demonstrate the achievement of saturation is invaluable since it explicates the parameters upon which saturation is contingent and indicates when a research project is likely to require a smaller or a larger sample [e.g. 29], specific numbers at which saturation was achieved within these projects cannot be routinely extrapolated for other projects. We concur with existing views [11, 36] that the consideration of the characteristics of the study at hand, such as the epistemological and theoretical approach, the nature of the phenomenon under investigation, the aims and scope of the study, the quality and richness of data, or the researcher’s experience and skills of conducting qualitative research, should be the primary guide in determining sample size and assessing its sufficiency.

Moreover, although numbers in qualitative research are not unimportant [61], sample size should not be considered alone but be embedded in the more encompassing examination of *data adequacy* [56, 57]. Erickson’s [62] dimensions of ‘evidentiary adequacy’ are useful here. He explains the concept in terms of adequate amounts of evidence, adequate variety in kinds of evidence, adequate interpretive status of evidence, adequate disconfirming evidence, and adequate discrepant case analysis. All dimensions might not be relevant across all qualitative research designs, but this illustrates the thickness of the concept of data adequacy, taking it beyond sample size.

The present research also demonstrated that sample sizes were commonly seen as ‘small’ and insufficient and discussed as limitation. Often unjustified (and in two cases incongruent with their own claims of saturation) these findings imply that sample size in qualitative health research is often adversely judged (or expected to be judged) against an implicit, yet omnipresent, quasi-quantitative standpoint. Indeed there were a few instances in our data where authors appeared, possibly in response to reviewers, to resist to some sort of quantification of their results. This implicit reference point became more apparent when authors discussed the threats deriving from an insufficient sample size. Whilst the concerns about internal validity might be legitimate to the extent that qualitative research projects, which are broadly related to realism, are set to examine phenomena in sufficient breadth and depth, the concerns around generalizability revealed a conceptualisation that is not compatible with purposive sampling. The limited potential for generalisation, as a result of a small sample size, was often discussed in nomothetic, statistical terms. Only occasionally was analytic or idiographic generalisation invoked to warrant the value of the study’s findings [5, 17].

### Strengths and limitations of the present study

We note, first, the limited number of health-related journals reviewed, so that only a ‘snapshot’ of qualitative health research has been captured. Examining additional disciplines (e.g. nursing sciences) as well as inter-disciplinary journals would add to the findings of this analysis. Nevertheless, our study is the first to provide some comparative insights on the basis of disciplines that are differently attached to the legacy of positivism and analysed literature published over a lengthy period of time (15 years). Guetterman [27] also examined health-related literature but this analysis was restricted to 26 most highly cited articles published over a period of five years whilst Carlsen and Glenton’s [22] study concentrated on focus groups health research. Moreover, although it was our intention to examine sample size justification in relation to the epistemological and theoretical positions of articles, this proved to be challenging largely due to absence of relevant information, or the difficulty into discerning clearly articles’ positions [63] and classifying them under specific approaches (e.g. studies often combined elements from different theoretical and epistemological traditions). We believe that such an analysis would yield useful insights as it links the methodological issue of sample size to the broader philosophical stance of the research. Despite these limitations, the analysis of the characterisation of sample size and of the threats seen to accrue from insufficient sample size, enriches our understanding of sample size (in)sufficiency argumentation by linking it to other features of the research. As the peer-review process becomes increasingly public, future research could usefully examine how reporting around sample size sufficiency and data adequacy might be influenced by the interactions between authors and reviewers.

## Conclusions

The past decade has seen a growing appetite in qualitative research for an evidence-based approach to sample size determination and to evaluations of the sufficiency of sample size. Despite the conceptual and methodological developments in the area, the findings of the present study confirm previous studies in concluding that appraisals of sample size sufficiency are either absent or poorly substantiated. To ensure and maintain high quality research that will encourage greater appreciation of qualitative work in health-related sciences [64], we argue that qualitative researchers should be more transparent and thorough in their evaluation of sample size as part of their appraisal of data adequacy. We would encourage the practice of appraising sample size sufficiency with close reference to the study at hand and would thus caution against responding to the growing methodological research in this area with a decontextualised application of sample size numerical guidelines, norms and principles. Although researchers might find sample size community norms serve as useful rules of thumb, we recommend methodological knowledge is used to critically consider how saturation and other parameters that affect sample size sufficiency pertain to the specifics of the particular project. Those reviewing papers have a vital role in encouraging transparent study-specific reporting. The review process should support authors to exercise nuanced judgments in decisions about sample size determination in the context of the range of factors that influence sample size sufficiency and the specifics of a particular study. In light of the growing methodological evidence in the area, transparent presentation of such evidence-based judgement is crucial and in time should surely obviate the seemingly routine practice of citing the ‘small’ size of qualitative samples among the study limitations.

## Additional Files

Additional File 1: Editorial positions on qualitative research and sample considerations (where available). (DOCX 12 kb)

Additional File 2: List of eligible articles included in the review (*N* = 214). (DOCX 38 kb)

Additional File 3: Data Extraction Form. (DOCX 15 kb)

Additional File 4: Citations used by articles to support their position on saturation. (DOCX 14 kb)

## Abbreviations

BJHP: British Journal of Health Psychology
BMJ: British Medical Journal
IPA: Interpretative Phenomenological Analysis
SHI: Sociology of Health & Illness

## Footnotes

2 The publication of qualitative studies in the BMJ was significantly reduced from 2012 onwards and this appears to coincide with the initiation of the BMJ Open to which qualitative studies were possibly directed.

1 A non-parametric test of difference for independent samples was performed since the variable *number of interviews* violated assumptions of normality according to the standardized scores of skewness and kurtosis (BMJ: *z* skewness = 3.23, *z* kurtosis = 1.52; BJHP: *z* skewness = 4.73, *z* kurtosis = 4.85; SHI: *z* skewness = 12.04, *z* kurtosis = 21.72) and the Shapiro-Wilk test of normality (*p* < .001).
