# Supplementary material for: Characterising and justifying sample size sufficiency in interview-based studies: systematic analysis of qualitative health research over a 15-year period
Source: BMC Med Res Methodol. 2018 Nov 21;18:148. doi: 10.1186/s12874-018-0594-7 (PMC6249736; doi:10.1186/s12874-018-0594-7)
Supplement: Supplementary file 1 — Editorial positions on qualitative research and sample considerations (where available). (DOCX 12 kb) [file 12874_2018_594_MOESM1_ESM.docx]

**Additional File 1**

Editorial positions on qualitative research and sample considerations (where available)

***BMJ***

The *British Medical Journal* explicitly states that it considers qualitative studies: “We are pleased to consider a wide range of study types, as long as the right design has been used to answer a relevant, important, and sufficiently original research question. These include randomised controlled trials of the effectiveness and safety of treatments and other clinical or healthcare interventions for patients with common diseases, studies on diagnostic tests, clinical and epidemiological observational studies (particularly on aetiology, prognosis, risk, and safety), evaluations of educational and quality improvement initiatives, **qualitative studies that help to explain why and how doctors and patients do things**, and systematic reviews of all of these study types.”

In relation to qualitative research it is noted that “if the sampling strategy was not clearly described or was driven by convenience rather than theory” publication in the journal is made impossible or unlikely.

<https://www.bmj.com/about-bmj/resources-authors/bmj-right-journal-my-research-article>

***BJHP***

The British Journal of Health Psychology explicitly invites empirical reports of both quantitative and qualitative research: “The types of paper invited are: papers reporting original empirical investigations, using either quantitative or qualitative methods, including reports of interventions in clinical and non-clinical populations”

Regarding sample considerations, the editorial policy of the journal states that: “In order to qualify for full review, papers must meet the following criteria…the methods and/or sample size are appropriate for the questions being addressed.”

(<https://onlinelibrary.wiley.com/page/journal/20448287/homepage/forauthors.html>)

***SHI***

The *Sociology of Health & Illness* does not express any particular position in relation to qualitative (or quantitative) research.

In relation to sample considerations, the journal instructs authors as follows: “The criteria for selecting the sample should be clearly described and justified, and the characteristics of the sample described.”

(<https://onlinelibrary.wiley.com/page/journal/14679566/homepage/forauthors.html>)
