# Supplementary material for: Characterising and justifying sample size sufficiency in interview-based studies: systematic analysis of qualitative health research over a 15-year period
Source: BMC Med Res Methodol. 2018 Nov 21;18:148. doi: 10.1186/s12874-018-0594-7 (PMC6249736; doi:10.1186/s12874-018-0594-7)
Supplement: Supplementary file 2 — List of eligible articles included in the review (N = 214). (DOCX 38 kb) [file 12874_2018_594_MOESM2_ESM.docx]

**Additional File 2**

List of eligible articles included in the review (*N* = 214)

| **British Medical Journal (BMJ)** | |
| --- | --- |
| BMJ01 | Stokes, T., Dixon-Woods, M., Windridge, K. C., & McKinley, R. K. (2003). Patients9 accounts of being removed from their general practitioner9s list: qualitative study. *Bmj, 326*(7402), 1316. |
| BMJ02 | Tarrant, C., Windridge, K., Boulton, M., Baker, R., & Freeman, G. (2003). Qualitative study of the meaning of personal care in general practice. *Bmj, 326*(7402), 1310. |
| BMJ03 | Davison, S. N., & Simpson, C. (2006). Hope and advance care planning in patients with end stage renal disease: qualitative interview study. *Bmj, 333*(7574), 886. |
| BMJ04 | Dixon-Woods, M., Jackson, C., Windridge, K. C., & Kenyon, S. (2006). Receiving a summary of the results of a trial: qualitative study of participants' views. *Bmj, 332*(7535), 206-210. |
| BMJ05 | Forrest, G., Plumb, C., Ziebland, S., & Stein, A. (2006). Breast cancer in the family—children's perceptions of their mother's cancer and its initial treatment: qualitative study. *Bmj, 332*(7548), 998-1003. |
| BMJ06 | Noble, S. I. R., Nelson, A., Turner, C., & Finlay, I. G. (2006). Acceptability of low molecular weight heparin thromboprophylaxis for inpatients receiving palliative care: qualitative study. *Bmj, 332*(7541), 577-580. |
| BMJ07 | Hendy, J., Fulop, N., Reeves, B. C., Hutchings, A., & Collin, S. (2007). Implementing the NHS information technology programme: qualitative study of progress in acute trusts. *Bmj, 334*(7608), 1360. |
| BMJ08 | Howerton, A., Byng, R., Campbell, J., Hess, D., Owens, C., & Aitken, P. (2007). Understanding help seeking behaviour among male offenders: qualitative interview study. *Bmj, 334*(7588), 303. |
| BMJ09 | Phillips, R., Amos, A., Ritchie, D., Cunningham-Burley, S., & Martin, C. (2007). Smoking in the home after the smoke-free legislation in Scotland: qualitative study. *Bmj, 335*(7619), 553. |
| BMJ10 | Carnes, D., Anwer, Y., Underwood, M., Harding, G., & Parsons, S. (2008). Influences on older people’s decision making regarding choice of topical or oral NSAIDs for knee pain: qualitative study. *Bmj, 336*(7636), 142-145. |
| BMJ11 | Rhodes, T., Simić, M., Baroš, S., Platt, L., & Žikić, B. (2008). Police violence and sexual risk among female and transvestite sex workers in Serbia: qualitative study. *Bmj, 337*, a811. |
| BMJ12 | Dowrick, C., Leydon, G. M., McBride, A., Howe, A., Burgess, H., Clarke, P., ... & Kendrick, T. (2009). Patients’ and doctors’ views on depression severity questionnaires incentivised in UK quality and outcomes framework: qualitative study. *Bmj, 338*, b663. |
| BMJ13 | Munday, D., Petrova, M., & Dale, J. (2009). Exploring preferences for place of death with terminally ill patients: qualitative study of experiences of general practitioners and community nurses in England. *Bmj,* *339*, b2391. |
| BMJ14 | Pasman, H. R. W., Rurup, M. L., Willems, D. L., & Onwuteaka-Philipsen, B. D. (2009). Concept of unbearable suffering in context of ungranted requests for euthanasia: qualitative interviews with patients and physicians. *BMJ, 339*, b4362. |
| BMJ15 | Selman, L., Higginson, I. J., Agupio, G., Dinat, N., Downing, J., Gwyther, L., ... & Panajatovic, B. (2009). Meeting information needs of patients with incurable progressive disease and their families in South Africa and Uganda: multicentre qualitative study. *Bmj, 338*, b1326. |
| BMJ16 | Chapple, A., & Ziebland, S. (2010). Viewing the body after bereavement due to a traumatic death: qualitative study in the UK. *Bmj, 340*, c2032. |
| BMJ17 | Leydon, G. M., Turner, S., Smith, H., & Little, P. (2010). Women’s views about management and cause of urinary tract infection: qualitative interview study. *Bmj, 340*, c279. |
| BMJ18 | Buiting, H. M., Rurup, M. L., Wijsbek, H., van Zuylen, L., & den Hartogh, G. (2011). Understanding provision of chemotherapy to patients with end stage cancer: qualitative interview study. *Bmj, 342*, d1933. |
| BMJ19 | Dowswell, G., Ismail, T., Greenfield, S., Clifford, S., Hancock, B., & Wilson, S. (2011). Men’s experience of erectile dysfunction after treatment for colorectal cancer: qualitative interview study. *Bmj, 343*, d5824. |
| BMJ20 | Iedema, R., Allen, S., Britton, K., Piper, D., Baker, A., Grbich, C., ... & Manias, E. (2011). Patients’ and family members’ views on how clinicians enact and how they should enact incident disclosure: the “100 patient stories” qualitative study. *Bmj, 343*, d4423. |
| BMJ21 | Owens, C., Owen, G., Belam, J., Lloyd, K., Rapport, F., Donovan, J., & Lambert, H. (2011). Recognising and responding to suicidal crisis within family and social networks: qualitative study. *Bmj, 343*, d5801. |
| **British Journal of Health Psychology** | |
| BJHP01 | Daniel, E., Kent, G., Binney, V., & Pagdin, J. (2005). Trying to do my best as a mother: decision‐making in families of children undergoing elective surgical treatment for short stature. *British journal of health psychology, 10*(1), 101-114. |
| BJHP02 | Watson, W. K., & Bell, N. J. (2005). Narratives of development, experiences of risk: Adult women's perspectives on relationships and safer sex. *British journal of health psychology, 10*(3), 311-327. |
| BJHP03 | Nicholls, K., & Ayers, S. (2007). Childbirth‐related post‐traumatic stress disorder in couples: A qualitative study. British journal of health psychology, 12(4), 491-509. |
| BJHP04 | Dovey‐Pearce, G., Doherty, Y., & May, C. (2007). The influence of diabetes upon adolescent and young adult development: a qualitative study. *British journal of health psychology, 12*(1), 75-91. |
| BJHP05 | Hale, S., Grogan, S., & Willott, S. (2007). Patterns of self‐referral in men with symptoms of prostate disease. *British journal of health psychology, 12*(3), 403-419. |
| BJHP06 | Hogg, N. M., Garratt, V., Shaw, S. K., & Tagney, J. (2007). It has certainly been good just to talk: An interpretative phenomenological analysis of coping with myocardial infarction. *British journal of health psychology, 12*(4), 651-662. |
| BJHP07 | Thornhill, K., Lyons, A. C., Nouwen, A., & Lip, G. Y. H. (2008). Experiences of living with congestive heart failure: a qualitative study. *British journal of health psychology, 13*(1), 155-175. |
| BJHP08 | Snelgrove, S., & Liossi, C. (2009). An interpretative phenomenological analysis of living with chronic low back pain. *British journal of health psychology, 14*(4), 735-749. |
| BJHP09 | Slade, P., Haywood, A., & King, H. (2009). A qualitative investigation of women's experiences of the self and others in relation to their menstrual cycle. *British journal of health psychology, 14*(1), 127-141. |
| BJHP10 | Piot‐Ziegler, C., Sassi, M. L., Raffoul, W., & Delaloye, J. F. (2010). Mastectomy, body deconstruction, and impact on identity: a qualitative study. *British journal of health psychology, 15*(3), 479-510. |
| BJHP11 | Beck, F., Gillison, F., & Standage, M. (2010). A theoretical investigation of the development of physical activity habits in retirement. *British journal of health psychology, 15*(3), 663-679. |
| BJHP12 | Hale, S., Grogan, S., & Willott, S. (2010). Male GPs' views on men seeking medical help: a qualitative study. *British journal of health psychology, 15*(4), 697-713. |
| BJHP13 | Dennison, L., Stanbrook, R., Moss‐Morris, R., Yardley, L., & Chalder, T. (2010). Cognitive behavioural therapy and psycho‐education for chronic fatigue syndrome in young people: reflections from the families' perspective. *British journal of health psychology, 15*(1), 167-183. |
| BJHP14 | Helweg‐Larsen, M., Tobias, M. R., & Cerban, B. M. (2010). Risk perception and moralization among smokers in the USA and Denmark: A qualitative approach. *British journal of health psychology, 15*(4), 871-886. |
| BJHP15 | Moore, S. N., Tapper, K., & Murphy, S. (2010). Feeding goals sought by mothers of 3–5‐year‐old children. *British journal of health psychology, 15*(1), 185-196. |
| BJHP16 | Southwell, O., & Fox, J. R. (2011). Maternal perceptions of overweight and obesity in children: a grounded theory study. *British journal of health psychology, 16*(3), 626-641. |
| BJHP17 | Bogosian, A., Moss‐Morris, R., Bishop, F. L., & Hadwin, J. (2011). How do adolescents adjust to their parent's multiple sclerosis?: An interview study. *British journal of health psychology, 16*(2), 430-444. |
| BJHP18 | Chambers, J. A., O’Carroll, R. E., Hamilton, B., Whittaker, J., Johnston, M., Sudlow, C., & Dennis, M. (2011). Adherence to medication in stroke survivors: a qualitative comparison of low and high adherers. *British journal of health psychology, 16*(3), 592-609. |
| BJHP19 | Devenney, R., & O’Neill, S. (2011). The experience of diabetic retinopathy: A qualitative study. *British journal of health psychology, 16*(4), 707-721. |
| BJHP20 | Ayers, S., Muller, I., Mahoney, L., & Seddon, P. (2011). Understanding needle‐related distress in children with cystic fibrosis. *British journal of health psychology, 16*(2), 329-343. |
| BJHP21 | Sawyer, A., Ayers, S., Smith, H., Sidibeh, L., Nyan, O., & Dale, J. (2011). Women's experiences of pregnancy, childbirth, and the postnatal period in the Gambia: a qualitative study. *British journal of health psychology, 16*(3), 528-541. |
| BJHP22 | Wadey, R., Evans, L., Hanton, S., & Neil, R. (2012). An examination of hardiness throughout the sport‐injury process: A qualitative follow‐up study. *British journal of health psychology, 17*(4), 872-893. |
| BJHP23 | Chambers, J. A., & Swanson, V. (2012). Stories of weight management: factors associated with successful and unsuccessful weight maintenance. *British journal of health psychology, 17*(2), 223-243. |
| BJHP24 | Delaney, M., Simpson, J., & Leroi, I. (2012). Perceptions of cause and control of impulse control behaviours in people with Parkinson's disease. *British journal of health psychology, 17*(3), 522-535. |
| BJHP25 | Fletcher, K. J., Nair, R. D., Macniven, J. A., Basu, S., & Byrne, P. (2012). An interpretative phenomenological analysis of the patient experience of awake craniotomy: Brain tumour diagnosis to discharge. *British journal of health psychology, 17*(4), 828-842. |
| BJHP26 | Vangeli, E., & West, R. (2012). Transition towards a ‘non‐smoker’ identity following smoking cessation: An interpretative phenomenological analysis. *British journal of health psychology, 17*(1), 171-184. |
| BJHP27 | Newby, K. V., Wallace, L. M., & French, D. P. (2012). How do young adults perceive the risk of chlamydia infection? A qualitative study. *British journal of health psychology, 17*(1), 144-154. |
| BJHP28 | McGowan, Y., Humphries, N., Burke, H., Conry, M., & Morgan, K. (2013). Through doctors' eyes: A qualitative study of hospital doctor perspectives on their working conditions. *British journal of health psychology, 18*(4), 874-891. |
| BJHP29 | Reme, S. E., Archer, N., & Chalder, T. (2013). Experiences of young people who have undergone the Lightning Process to treat chronic fatigue syndrome/myalgic encephalomyelitis–a qualitative study. *British journal of health psychology, 18*(3), 508-525. |
| BJHP30 | Hynes, L., Byrne, M., Casey, D., Dinneen, S. F., & O'hara, M. C. (2015). ‘It makes a difference, coming here’: A qualitative exploration of clinic attendance among young adults with type 1 diabetes. *British journal of health psychology, 20*(4), 842-858. |
| BJHP31 | Peters, S., Goldthorpe, J., McElroy, C., King, E., Javidi, H., Tickle, M., & Aggarwal, V. R. (2015). Managing chronic orofacial pain: A qualitative study of patients', doctors', and dentists' experiences. *British journal of health psychology, 20*(4), 777-791. |
| BJHP32 | Bond, M., Garside, R., & Hyde, C. (2015). A crisis of visibility: The psychological consequences of false‐positive screening mammograms, an interview study. *British journal of health psychology, 20*(4), 792-806. |
| BJHP33 | Tiwana, R., Rowland, J., Fincher, M., Raza, K., & Stack, R. J. (2015). Social interactions at the onset of rheumatoid arthritis and their influence on help‐seeking behaviour: A qualitative exploration. *British journal of health psychology, 20*(3), 648-661. |
| BJHP34 | Murray, C. D., Turner, A., Rehan, C., & Kovacs, T. (2015). Satisfaction following immediate breast reconstruction: Experiences in the early post‐operative stage. *British journal of health psychology, 20*(3), 579-593. |
| BJHP35 | Tang, J., Abraham, C., Stamp, E., & Greaves, C. (2015). How can weight‐loss app designers' best engage and support users? A qualitative investigation. *British journal of health psychology, 20*(1), 151-171. |
| BJHP36 | Watson, L. A., Baker, M. C., & Chadwick, P. M. (2016). Kids just wanna have fun: Children's experiences of a weight management programme. *British journal of health psychology, 21*(2), 407-420. |
| BJHP37 | Damman, O. C., Bogaerts, N. M., Dongen, D., & Timmermans, D. R. (2016). Barriers in using cardiometabolic risk information among consumers with low health literacy. *British journal of health psychology, 21*(1), 135-156. |
| BJHP38 | Walton, P. D., & French, D. P. (2016). What do people think about running barefoot/with minimalist footwear? A thematic analysis. *British journal of health psychology, 21*(2), 451-468. |
| BJHP39 | Gotts, Z. M., Newton, J. L., Ellis, J. G., & Deary, V. (2016). The experience of sleep in chronic fatigue syndrome: A qualitative interview study with patients. *British journal of health psychology, 21*(1), 71-92. |
| BJHP40 | Zalai, D., Carney, C. E., Sherman, M., Shapiro, C. M., & McShane, K. (2016). Fatigue in chronic hepatitis C infection: Understanding patients' experience from a cognitive‐behavioural perspective. *British journal of health psychology, 21*(1), 157-172. |
| BJHP41 | Coleman, B., Ellis‐Caird, H., McGowan, J., & Benjamin, M. J. (2016). How sickle cell disease patients experience, understand and explain their pain: An Interpretative Phenomenological Analysis study. *British journal of health psychology, 21*(1), 190-203. |
| BJHP42 | Finlay, K. A., & Elander, J. (2016). Reflecting the transition from pain management services to chronic pain support group attendance: An interpretative phenomenological analysis. *British journal of health psychology, 21*(3), 660-676. |
| BJHP43 | Atkinson, L., Shaw, R. L., & French, D. P. (2016). Is pregnancy a teachable moment for diet and physical activity behaviour change? An interpretative phenomenological analysis of the experiences of women during their first pregnancy. *British journal of health psychology, 21*(4), 842-858. |
| BJHP44 | Chisholm, A., Nelson, P. A., Pearce, C. J., Keyworth, C., Griffiths, C. E., Cordingley, L., & Bundy, C. (2016). The role of personal models in clinical management: Exploring health care providers’ beliefs about psoriasis. *British journal of health psychology, 21*(1), 114-134. |
| BJHP45 | Merritt, C. J., Zoysa, N., & Hutton, J. M. (2017). A qualitative study of younger men's experience of heart attack (myocardial infarction). *British journal of health psychology, 22*, 589–608. |
| BJHP46 | Tarrant, M., Khan, S. S., Farrow, C. V., Shah, P., Daly, M., & Kos, K. (2017). Patient experiences of a bariatric group programme for managing obesity: A qualitative interview study. *British journal of health psychology, 22*(1), 77-93. |
| BJHP47 | Schütze, R., Rees, C., Slater, H., Smith, A., & O'Sullivan, P. (2017). ‘I call it stinkin’ thinkin’’: A qualitative analysis of metacognition in people with chronic low back pain and elevated catastrophizing. *British journal of health psychology, 22*, 463–480 |
| BJHP48 | Moon, Z., Moss‐Morris, R., Hunter, M. S., & Hughes, L. D. (2017). Understanding tamoxifen adherence in women with breast cancer: A qualitative study. *British journal of health psychology, 22*(4), 978-997. |
| BJHP49 | Sibelli, A., Chalder, T., Everitt, H., Workman, P., Bishop, F. L., & Moss‐Morris, R. (2017). The role of high expectations of self and social desirability in emotional processing in individuals with irritable bowel syndrome: A qualitative study. *British journal of health psychology, 22*(4), 737-762. |
| BJHP50 | Picariello, F., Ali, S., Foubister, C., & Chalder, T. (2017). ‘It feels sometimes like my house has burnt down, but I can see the sky’: A qualitative study exploring patients’ views of cognitive behavioural therapy for chronic fatigue syndrome. *British Journal of Health Psychology, 22*, 383–413. |
| BJHP51 | Marcu, A., Black, G., Vedsted, P., Lyratzopoulos, G., & Whitaker, K. L. (2017). Educational differences in responses to breast cancer symptoms: A qualitative comparative study. *British journal of health psychology, 22*(1), 26-41. |
| BJHP52 | Taylor, E. C., O'Neill, M., Hughes, L. D., Carroll, S., & Moss‐Morris, R. (2017). ‘It's like a frog leaping about in your chest’: Illness and treatment perceptions in persistent atrial fibrillation. *British journal of health psychology, 23*, 3–21. |
| BJHP53 | Newby, K. V., Cook, C., Meisel, S. F., Webb, T. L., Fisher, B., & Fisher, A. (2017). Young people's beliefs about the risk of bowel cancer and its link with physical activity. *British Journal of Health Psychology, 22*, 449–462. |
| **Sociology of Health & Illness** | |
| SHI01 | Charles‐Jones, H., Latimer, J., & May, C. (2003). Transforming general practice: the redistribution of medical work in primary care. *Sociology of health & illness, 25*(1), 71-92. |
| SHI02 | Corrigan, O. (2003). Empty ethics: the problem with informed consent. *Sociology of health & illness, 25*(7), 768-792. |
| SHI03 | Henwood, F., Wyatt, S., Hart, A., & Smith, J. (2003). ‘Ignorance is bliss sometimes’: constraints on the emergence of the ‘informed patient’ in the changing landscapes of health information. *Sociology of health & illness, 25*(6), 589-607. |
| SHI04 | Lowton, K., & Gabe, J. (2003). Life on a slippery slope: perceptions of health in adults with cystic fibrosis. *Sociology of health & illness, 25*(4), 289-319. |
| SHI05 | Murtagh, M. J., & Hepworth, J. (2003). Menopause as a long‐term risk to health: implications of general practitioner accounts of prevention for women's choice and decision‐making. *Sociology of health & illness, 25*(2), 185-207. |
| SHI06 | Redley, M. (2003). Towards a new perspective on deliberate self‐harm in an area of multiple deprivation. *Sociology of health & illness, 25*(4), 348-372. |
| SHI07 | Welsh, S., Kelner, M., Wellman, B., & Boon, H. (2004). Moving forward? Complementary and alternative practitioners seeking self‐regulation. *Sociology of health & illness, 26*(2), 216-241. |
| SHI08 | Walls, P., & Williams, R. (2004). Accounting for Irish Catholic ill health in Scotland: a qualitative exploration of some links between ‘religion’, class and health. *Sociology of health & illness, 26*(5), 527-556. |
| SHI09 | Rosengarten, M., Imrie, J., Flowers, P., Davis, M. D., & Hart, G. (2004). After the euphoria: HIV medical technologies from the perspective of their prescribers. *Sociology of health & illness, 26*(5), 575-596. |
| SHI10 | Gregory, S. (2005). Living with chronic illness in the family setting. *Sociology of health & illness, 27*(3), 372-392. |
| SHI11 | Rafalovich, A. (2005). Exploring clinician uncertainty in the diagnosis and treatment of attention deficit hyperactivity disorder. *Sociology of health & illness, 27*(3), 305-323. |
| SHI12 | Ville, I. (2005). Biographical work and returning to employment following a spinal cord injury. *Sociology of health & illness, 27*(3), 324-350. |
| SHI13 | Liamputtong, P. (2005). Birth and social class: Northern Thai women's lived experiences of caesarean and vaginal birth. *Sociology of health & illness, 27*(2), 243-270. |
| SHI14 | Murray, S. F., & Elston, M. A. (2005). The promotion of private health insurance and its implications for the social organisation of healthcare: a case study of private sector obstetric practice in Chile. *Sociology of health & illness, 27*(6), 701-721. |
| SHI15 | Lupton, D. A. (2005). Lay discourses and beliefs related to food risks: an Australian perspective. *Sociology of health & illness, 27*(4), 448-467. |
| SHI16 | Haddow, G. (2005). The phenomenology of death, embodiment and organ transplantation. *Sociology of health & illness, 27*(1), 92-113. |
| SHI17 | Barnard, M. (2005). Discomforting research: Colliding moralities and looking for ‘truth’ in a study of parental drug problems. *Sociology of health & illness, 27*(1), 1-19. |
| SHI18 | Harden, J. (2005). Parenting a young person with mental health problems: Temporal disruption and reconstruction. *Sociology of health & illness, 27*(3), 351-371. |
| SHI19 | Marston, C. (2005). What is heterosexual coercion? Interpreting narratives from young people in Mexico City. *Sociology of health & illness, 27*(1), 68-91. |
| SHI20 | Nettleton, S., Burrows, R., & O'Malley, L. (2005). The mundane realities of the everyday lay use of the internet for health, and their consequences for media convergence. *Sociology of health & illness, 27*(7), 972-992. |
| SHI21 | Armstrong, D., & Ogden, J. (2006). The role of etiquette and experimentation in explaining how doctors change behaviour: a qualitative study. *Sociology of health & illness, 28*(7), 951-968. |
| SHI22 | Coxhead, L., & Rhodes, T. (2006). Accounting for risk and responsibility associated with smoking among mothers of children with respiratory illness. *Sociology of health & illness, 28*(1), 98-121. |
| SHI23 | Davis, M., Hart, G., Bolding, G., Sherr, L., & Elford, J. (2006). E‐dating, identity and HIV prevention: theorising sexualities, risk and network society. *Sociology of health & illness, 28*(4), 457-478. |
| SHI24 | Hallowell, N., Arden‐Jones, A., Eeles, R., Foster, C., Lucassen, A., Moynihan, C., & Watson, M. (2006). Guilt, blame and responsibility: men's understanding of their role in the transmission of BRCA1/2 mutations within their family. *Sociology of health & illness, 28*(7), 969-988. |
| SHI25 | Roderick, M. (2006). Adding insult to injury: workplace injury in English professional football. *Sociology of health & illness, 28*(1), 76-97. |
| SHI26 | Shim, J. K., Russ, A. J., & Kaufman, S. R. (2006). Risk, life extension and the pursuit of medical possibility. Sociology of health & illness, 28(4), 479-502. |
| SHI27 | Sointu, E. (2006). The search for wellbeing in alternative and complementary health practices. *Sociology of health & illness, 28*(3), 330-349. |
| SHI28 | Williams, C. (2006). Dilemmas in fetal medicine: premature application of technology or responding to women's choice?. *Sociology of health & illness, 28*(1), 1-20. |
| SHI29 | Wainwright, S. P., Williams, C., Michael, M., Farsides, B., & Cribb, A. (2006). Ethical boundary‐work in the embryonic stem cell laboratory. *Sociology of health & illness, 28*(6), 732-748. |
| SHI30 | Biddle, L., Donovan, J., Sharp, D., & Gunnell, D. (2007). Explaining non‐help‐seeking amongst young adults with mental distress: a dynamic interpretive model of illness behaviour. *Sociology of health & illness,* *29*(7), 983-1002. |
| SHI31 | Payne, J. (2007). Women drug users in North Cumbria: what influences initiation into heroin in this non‐urban setting?. *Sociology of health & illness, 29*(5), 633-655. |
| SHI32 | Jackson, S., & Scambler, G. (2007). Perceptions of evidence‐based medicine: Traditional acupuncturists in the UK and resistance to biomedical modes of evaluation. *Sociology of health & illness, 29*(3), 412-429. |
| SHI33 | Frost, J., Bradley, H., Levitas, R., Smith, L., & Garcia, J. (2007). The loss of possibility: scientisation of death and the special case of early miscarriage. *Sociology of health & illness, 29*(7), 1003-1022. |
| SHI34 | Feldmann, C. T., Bensing, J. M., De Ruijter, A., & Boeije, H. R. (2007). Afghan refugees and their general practitioners in The Netherlands: to trust or not to trust?. *Sociology of health & illness, 29*(4), 515-535. |
| SHI35 | Dolan, A. (2007). ‘Good luck to them if they can get it’: exploring working class men's understandings and experiences of income inequality and material standards. Sociology of health & illness, 29(5), 711-729. |
| SHI36 | Dew, K., Morgan, S., Dowell, A., McLeod, D., Bushnell, J., & Collings, S. (2007). ‘It puts things out of your control’: fear of consequences as a barrier to patient disclosure of mental health issues to general practitioners. *Sociology of health & illness, 29*(7), 1059-1074. |
| SHI37 | Calnan, M., Wainwright, D., O’Neill, C., Winterbottom, A., & Watkins, C. (2007). Illness action rediscovered: a case study of upper limb pain. *Sociology of health & illness, 29*(3), 321-346. |
| SHI38 | Broom, A., & Tovey, P. (2007). Therapeutic pluralism? Evidence, power and legitimacy in UK cancer services. *Sociology of health & illness, 29*(4), 551-569. |
| SHI39 | Crawford, P., & Brown, B. (2008). Soft authority: ecologies of infection management in the working lives of modern matrons and infection control staff. *Sociology of health & illness, 30*(5), 756-771. |
| SHI40 | Hashiloni‐Dolev, Y., & Weiner, N. (2008). New reproductive technologies, genetic counselling and the standing of the fetus: views from Germany and Israel. *Sociology of health & illness, 30*(7), 1055-1069. |
| SHI41 | Theberge, N. (2008). The integration of chiropractors into healthcare teams: a case study from sport medicine. *Sociology of health & illness, 30*(1), 19-34. |
| SHI42 | Sered, S., & Agigian, A. (2008). Holistic sickening: breast cancer and the discursive worlds of complementary and alternative practitioners. *Sociology of health & illness, 30*(4), 616-631. |
| SHI43 | Sanders, T., & Harrison, S. (2008). Professional legitimacy claims in the multidisciplinary workplace: the case of heart failure care. *Sociology of health & illness, 30*(2), 289-308. |
| SHI44 | Pietilä, I., & Rytkönen, M. (2008). ‘Health is not a man's domain’: lay accounts of gender difference in life‐expectancy in Russia. *Sociology of health & illness, 30*(7), 1070-1085. |
| SHI45 | Owens, C., Lambert, H., Lloyd, K., & Donovan, J. (2008). Tales of biographical disintegration: how parents make sense of their sons’ suicides. *Sociology of health & Illness, 30*(2), 237-254. |
| SHI46 | Noone, J. H., & Stephens, C. (2008). Men, masculine identities, and health care utilisation. *Sociology of health & illness, 30*(5), 711-725. |
| SHI47 | Nettleton, S., Burrows, R., & Watt, I. (2008). Regulating medical bodies? The consequences of the ‘modernisation’ of the NHS and the disembodiment of clinical knowledge. *Sociology of health & illness,* *30*(3), 333-348. |
| SHI48 | MacRae, H. (2008). ‘Making the best you can of it’: living with early‐stage Alzheimer's disease. *Sociology of health & illness, 30*(3), 396-412. |
| SHI49 | Järvinen, M. (2008). Approaches to methadone treatment: harm reduction in theory and practice. *Sociology of health & illness, 30*(7), 975-991. |
| SHI50 | Wainwright, S. P., Michael, M., & Williams, C. (2008). Shifting paradigms? Reflections on regenerative medicine, embryonic stem cells and pharmaceuticals. *Sociology of health & illness, 30*(6), 959-974. |
| SHI51 | Thorpe, R. D. (2009). ‘Doing’ chronic illness? Complementary medicine use among people living with HIV/AIDS in Australia. *Sociology of health & illness, 31*(3), 375-389. |
| SHI52 | Simić, M., & Rhodes, T. (2009). Violence, dignity and HIV vulnerability: street sex work in Serbia. *Sociology of health & illness, 31*(1), 1-16. |
| SHI53 | Rozario, P. A., & Derienzis, D. (2009). ‘So forget how old I am!’ Examining age identities in the face of chronic conditions. *Sociology of health & illness, 31*(4), 540-553. |
| SHI54 | Locock, L., Ziebland, S., & Dumelow, C. (2009). Biographical disruption, abruption and repair in the context of motor neurone disease. *Sociology of health & illness, 31*(7), 1043-1058. |
| SHI55 | Kelly, S. E. (2009). Choosing not to choose: reproductive responses of parents of children with genetic conditions or impairments. *Sociology of Health & Illness, 31*(1), 81-97. |
| SHI56 | Harris, M. (2009). Troubling biographical disruption: narratives of unconcern about hepatitis C diagnosis. *Sociology of health & illness, 31*(7), 1028-1042. |
| SHI57 | Hurd Clarke, L., & Korotchenko, A. (2009). Older women and suntanning: the negotiation of health and appearance risks. *Sociology of health & illness, 31*(5), 748-761. |
| SHI58 | Keeley, B., Wright, L., & Condit, C. M. (2009). Functions of health fatalism: fatalistic talk as face saving, uncertainty management, stress relief and sense making. *Sociology of health & illness, 31*(5), 734-747. |
| SHI59 | Williams, B., Woodby, L., & Drentea, P. (2010). Ethical capital: ‘What’s a poor man got to leave?’ *Sociology of health & illness, 32*(6), 880–897. |
| SHI60 | Polich, G., Dole, C., & Kaptchuk, T. J. (2010). The need to act a little more ‘scientific’: biomedical researchers investigating complementary and alternative medicine. *Sociology of health & illness, 32*(1), 106-122. |
| SHI61 | Markens, S., Browner, C. H., & Mabel Preloran, H. (2010). Interrogating the dynamics between power, knowledge and pregnant bodies in amniocentesis decision making. *Sociology of health & illness, 32*(1), 37-56. |
| SHI62 | Lehoux, P., Denis, J. L., Rock, M., Hivon, M., & Tailliez, S. (2010). How medical specialists appraise three controversial health innovations: scientific, clinical and social arguments. *Sociology of health & illness, 32*(1), 123-139. |
| SHI63 | Kokanovic, R., May, C., Dowrick, C., Furler, J., Newton, D., & Gunn, J. (2010). Negotiations of distress between East Timorese and Vietnamese refugees and their family doctors in Melbourne. *Sociology of health & illness, 32*(4), 511-527. |
| SHI64 | Kinnunen, T. (2010). ‘A second youth’: pursuing happiness and respectability through cosmetic surgery in Finland. *Sociology of health & illness, 32*(2), 258-271. |
| SHI65 | Katainen, A. (2010). Social class differences in the accounts of smoking–striving for distinction?. *Sociology of health & illness, 32*(7), 1087-1101. |
| SHI66 | Jaye, C., & Fitzgerald, R. (2010). The lived political economy of occupational overuse syndrome among New Zealand workers. *Sociology of health & illness, 32*(7), 1010-1025. |
| SHI67 | Fishman, J. R., Settersten Jr, R. A., & Flatt, M. A. (2010). In the vanguard of biomedicine? The curious and contradictory case of anti‐ageing medicine. *Sociology of health & illness, 32*(2), 197-210. |
| SHI68 | Fallon, D. (2010). Accessing emergency contraception: the role of friends in the adolescent experience. *Sociology of health & illness, 32*(5), 677-694. |
| SHI69 | Devries, K. M., & Free, C. (2010). ‘I told him not to use condoms’: masculinities, femininities and sexual health of Aboriginal Canadian young people. *Sociology of health & illness, 32*(6), 827-842. |
| SHI70 | Carter, S. K. (2010). Beyond control: Body and self in women’s childbearing narratives. *Sociology of health & illness, 32*(7), 993-1009. |
| SHI71 | Brooks, A. T. (2010). Aesthetic anti‐ageing surgery and technology: women’s friend or foe?. *Sociology of health & illness, 32*(2), 238-257. |
| SHI72 | Bell, K., McCullough, L., Salmon, A., & Bell, J. (2010). ‘Every space is claimed’: Smokers’ experiences of tobacco denormalisation. *Sociology of health & illness, 32*(6), 914-929. |
| SHI73 | Balfe, M., & Brugha, R. (2010). Disclosure of STI testing activities by young adults: the influence of emotions and social networks. *Sociology of health & illness, 32*(7), 1041-1058. |
| SHI74 | Shoveller, J. A., Knight, R., Johnson, J., Oliffe, J. L., & Goldenberg, S. (2010). ‘Not the swab!’Young men’s experiences with STI testing. *Sociology of health & illness, 32*(1), 57-73. |
| SHI75 | Bloor, M. (2011). An essay on ‘health capital’and the Faustian bargains struck by workers in the globalised shipping industry. *Sociology of health & illness, 33*(7), 973-986. |
| SHI76 | Brown, P. R., Alaszewski, A., Swift, T., & Nordin, A. (2011). Actions speak louder than words: the embodiment of trust by healthcare professionals in gynae‐oncology. *Sociology of health & illness, 33*(2), 280-295. |
| SHI77 | Dyson, S. M., Atkin, K., Culley, L. A., Dyson, S. E., & Evans, H. (2011). Sickle cell, habitual dys‐positions and fragile dispositions: young people with sickle cell at school. *Sociology of health & illness, 33*(3), 465-483. |
| SHI78 | Frith, L., Jacoby, A., & Gabbay, M. (2011). Ethical boundary‐work in the infertility clinic. *Sociology of health & illness, 33*(4), 570-585. |
| SHI79 | Green, J., Durand, M. A., Hutchings, A., & Black, N. (2011). Modernisation as a professionalising strategy: the case of critical care in England. *Sociology of health & illness, 33*(6), 819-836. |
| SHI80 | Gundersen, T. (2011). ‘One wants to know what a chromosome is’: the internet as a coping resource when adjusting to life parenting a child with a rare genetic disorder. *Sociology of health & illness, 33*(1), 81-95. |
| SHI81 | Mitchell, K. R., Wellings, K., Nazareth, I., King, M., Mercer, C. H., & Johnson, A. M. (2011). Scripting sexual function: a qualitative investigation. *Sociology of health & illness, 33*(4), 540-553. |
| SHI82 | Nettleton, S., Neale, J., & Pickering, L. (2011). ‘I don’t think there’s much of a rational mind in a drug addict when they are in the thick of it’: towards an embodied analysis of recovering heroin users. *Sociology of health & illness, 33*(3), 341-355. |
| SHI83 | Pickersgill, M. (2011). ‘Promising’therapies: neuroscience, clinical practice, and the treatment of psychopathy. *Sociology of health & illness, 33*(3), 448-464. |
| SHI84 | Robinson, J., Ritchie, D., Amos, A., Greaves, L., & Cunningham‐Burley, S. (2011). Volunteered, negotiated, enforced: family politics and the regulation of home smoking. *Sociology of health & illness, 33*(1), 66-80. |
| SHI85 | Sanderson, T., Calnan, M., Morris, M., Richards, P., & Hewlett, S. (2011). Shifting normalities: interactions of changing conceptions of a normal life and the normalisation of symptoms in rheumatoid arthritis. *Sociology of health & illness, 33*(4), 618-633. |
| SHI86 | Sointu, E. (2011). Detraditionalisation, gender and alternative and complementary medicines. Sociology of health & illness, 33(3), 356-371. |
| SHI87 | Wong, W. C., Holroyd, E., & Bingham, A. (2011). Stigma and sex work from the perspective of female sex workers in Hong Kong. *Sociology of health & illness, 33*(1), 50-65. |
| SHI88 | Blackman, T., Harrington, B., Elliott, E., Greene, A., Hunter, D. J., Marks, L., ... & Williams, G. (2012). Framing health inequalities for local intervention: comparative case studies. *Sociology of health & illness, 34*(1), 49-63. |
| SHI89 | Francis, A. (2012). Stigma in an era of medicalisation and anxious parenting: how proximity and culpability shape middle‐class parents’ experiences of disgrace. *Sociology of health & illness, 34*(6), 927-942. |
| SHI90 | Frohlich, K. L., Mykhalovskiy, E., Poland, B. D., Haines‐Saah, R., & Johnson, J. (2012). Creating the socially marginalised youth smoker: the role of tobacco control. *Sociology of health & illness, 34*(7), 978-993. |
| SHI91 | Funk, L. M., Stajduhar, K. I., Robin Cohen, S., Heyland, D. K., & Williams, A. (2012). Legitimising and rationalising in talk about satisfaction with formal healthcare among bereaved family members. *Sociology of health & illness, 34*(7), 1010-1024. |
| SHI92 | del Mar García‐Calvente, M., Hidalgo‐Ruzzante, N., del Río‐Lozano, M., Marcos‐Marcos, J., Martínez‐Morante, E., Maroto‐Navarro, G., ... & Gil‐García, E. (2012). Exhausted women, tough men: a qualitative study on gender differences in health, vulnerability and coping with illness in Spain. *Sociology of health & illness, 34*(6), 911-926. |
| SHI93 | Gillespie, C. (2012). The experience of risk as ‘measured vulnerability’: health screening and lay uses of numerical risk. *Sociology of health & illness, 34*(2), 194-207. |
| SHI94 | Haimes, E., Taylor, K., & Turkmendag, I. (2012). Eggs, ethics and exploitation? Investigating women’s experiences of an egg sharing scheme. *Sociology of health & illness, 34*(8), 1199-1214. |
| SHI95 | Håland, E. (2012). Introducing the electronic patient record (EPR) in a hospital setting: boundary work and shifting constructions of professional identities. *Sociology of health & illness, 34*(5), 761-775. |
| SHI96 | Johnson, J. L., Oliffe, J. L., Kelly, M. T., Galdas, P., & Ogrodniczuk, J. S. (2012). Men’s discourses of help‐seeking in the context of depression. *Sociology of health & illness, 34*(3), 345-361. |
| SHI97 | Knight, R., Shoveller, J. A., Oliffe, J. L., Gilbert, M., Frank, B., & Ogilvie, G. (2012). Masculinities, ‘guy talk’ and ‘manning up’: a discourse analysis of how young men talk about sexual health. *Sociology of health & illness, 34*(8), 1246-1261. |
| SHI98 | Koo, K. (2012). Carers’ representations of affective mental disorders in British Chinese communities. *Sociology of health & illness, 34*(8), 1140-1155. |
| SHI99 | Lie, M., May, C., Kelly, T., & Robson, S. (2012). ‘Let the computer choose?’: the experience of participants in a randomised preference trial of medical versus surgical termination of pregnancy. *Sociology of health & illness, 34*(5), 746-760. |
| SHI100 | Lovelock, K. (2012). The injured and diseased farmer: Occupational health, embodiment and technologies of harm and care. *Sociology of health & illness, 34*(4), 576-590. |
| SHI101 | Montgomery, C. M. (2012). Protocols and participatory democracy in a ‘North–South’ product development partnership. *Sociology of health & illness, 34*(7), 1053-1069. |
| SHI102 | Mozersky, J. (2012). Who’s to blame? Accounts of genetic responsibility and blame among Ashkenazi Jewish women at risk of BRCA breast cancer. *Sociology of health & illness, 34*(5), 776-790. |
| SHI103 | Prior, S. (2012). Overcoming stigma: How young people position themselves as counselling service users. *Sociology of health & illness, 34*(5), 697-713. |
| SHI104 | Ridge, D., & Ziebland, S. (2012). Understanding depression through a ‘coming out’ framework. *Sociology of health & illness, 34*(5), 730-745. |
| SHI105 | Shostak, S., & Fox, N. S. (2012). Forgetting and remembering epilepsy: collective memory and the experience of illness. *Sociology of health & illness, 34*(3), 362-378. |
| SHI106 | Szewczuk, E. (2012). Age‐related infertility: a tale of two technologies. *Sociology of health & illness, 34*(3), 429-443. |
| SHI107 | Venn, S., & Arber, S. (2012). Understanding older peoples’ decisions about the use of sleeping medication: issues of control and autonomy. *Sociology of health & illness, 34*(8), 1215-1229. |
| SHI108 | Waitzkin, H., Yager, J., & Santos, R. (2012). Advancing the business creed? The framing of decisions about public sector managed care. *Sociology of health & illness, 34*(1), 31-48. |
| SHI109 | Waldby, C., & Carroll, K. (2012). Egg donation for stem cell research: ideas of surplus and deficit in Australian IVF patients’ and reproductive donors’ accounts. *Sociology of health & illness, 34*(4), 513-528. |
| SHI110 | Rhodes, T., Harris, M., & Martin, A. (2013). Negotiating access to medical treatment and the making of patient citizenship: the case of hepatitis C treatment. *Sociology of health & illness, 35*(7), 1023-1044. |
| SHI111 | Savage, M., Dumas, A., & Stuart, S. A. (2013). Fatalism and short‐termism as cultural barriers to cardiac rehabilitation among underprivileged men. *Sociology of health & illness, 35*(8), 1211-1226. |
| SHI112 | Singh, I. (2013). Brain talk: power and negotiation in children’s discourse about self, brain and behaviour. *Sociology of health & illness, 35*(6), 813-827. |
| SHI113 | Smith, D., & Ruston, A. (2013). ‘If you feel that nobody wants you you'll withdraw into your own’: Gypsies/Travellers, networks and healthcare utilisation. *Sociology of health & illness, 35*(8), 1196-1210. |
| SHI114 | Broom, A., & Kirby, E. (2013). The end of life and the family: hospice patients’ views on dying as relational. *Sociology of health & illness, 35*(4), 499-513. |
| SHI115 | Buchman, D. Z., Borgelt, E. L., Whiteley, L., & Illes, J. (2013). Neurobiological narratives: Experiences of mood disorder through the lens of neuroimaging. *Sociology of health & illness, 35*(1), 66-81. |
| SHI116 | Chase, E. (2013). Security and subjective wellbeing: the experiences of unaccompanied young people seeking asylum in the UK. *Sociology of health & illness, 35*(6), 858-872. |
| SHI117 | Kitzinger, J., & Kitzinger, C. (2013). The ‘window of opportunity’ for death after severe brain injury: family experiences. *Sociology of health & illness, 35*(7), 1095-1112. |
| SHI118 | Kokanovic, R., Bendelow, G., & Philip, B. (2013). Depression: the ambivalence of diagnosis. *Sociology of health & illness, 35*(3), 377-390. |
| SHI119 | Lupton, D., & Schmied, V. (2013). Splitting bodies/selves: women’s concepts of embodiment at the moment of birth. *Sociology of health & illness, 35*(6), 828-841. |
| SHI120 | MacBride‐Stewart, S. (2013). The effort to control time in the ‘new’ general practice. *Sociology of health & illness, 35*(4), 560-574. |
| SHI121 | Macdonald, S., Watt, G., & Macleod, U. (2013). In search of the cancer candidate: can lay epidemiology help?. *Sociology of health & illness, 35*(4), 575-591. |
| SHI122 | Murdoch, J., Salter, C., Cross, J., Smith, J., & Poland, F. (2013). Resisting medications: moral discourses and performances in illness narratives. *Sociology of health & illness, 35*(3), 449-464. |
| SHI123 | Pedersen, W., & Sandberg, S. (2013). The medicalisation of revolt: a sociological analysis of medical cannabis users. *Sociology of health & illness, 35*(1), 17-32. |
| SHI124 | Jylhänkangas, L., Smets, T., Cohen, J., Utriainen, T., & Deliens, L. (2014). Descriptions of euthanasia as social representations: Comparing the views of Finnish physicians and religious professionals. *Sociology of health & illness, 36*(3), 354-368. |
| SHI125 | Rousseau, N., Steele, J., May, C., & Exley, C. (2014). ‘Your whole life is lived through your teeth’: biographical disruption and experiences of tooth loss and replacement. *Sociology of health & illness, 36*(3), 462-476. |
| SHI126 | Will, C. M., & Weiner, K. (2014). Sustained multiplicity in everyday cholesterol reduction: repertoires and practices in talk about ‘healthy living’. *Sociology of health & illness, 36*(2), 291-304. |
| SHI127 | Adams, W. E., Todorova, I. L., Guzzardo, M. T., & Falcón, L. M. (2015). ‘The problem here is that they want to solve everything with pills’: medication use and identity among Mainland Puerto Ricans. *Sociology of health & illness, 37*(6), 904-919. |
| SHI128 | Järvinen, M., & Ravn, S. (2015). Explanations and expectations: Drug narratives among young cannabis users in treatment. *Sociology of health & illness, 37*(6), 870-887. |
| SHI129 | Jordan, J., Price, J., & Prior, L. (2015). Disorder and disconnection: parent experiences of liminality when caring for their dying child. *Sociology of health & illness, 37*(6), 839-855. |
| SHI130 | Lerum, S. V., Solbrække, K. N., Holmøy, T., & Frich, J. C. (2015). Unstable terminality: negotiating the meaning of chronicity and terminality in motor neurone disease. *Sociology of health & illness, 37*(1), 81-96. |
| SHI131 | Monaghan, L. F., & Gabe, J. (2015). Chronic illness as biographical contingency? Young people's experiences of asthma. *Sociology of health & illness, 37*(8), 1236-1253. |
| SHI132 | Ward, P. R., Coffey, C., & Meyer, S. (2015). Trust, choice and obligation: a qualitative study of enablers of colorectal cancer screening in South Australia. *Sociology of health & illness, 37*(7), 988-1006. |
| SHI133 | Wilhelmsen, T., & Nilsen, R. D. (2015). Parents' experiences of diagnostic processes of young children in Norwegian day‐care institutions. *Sociology of health & illness, 37*(2), 241-254. |
| SHI134 | Aasbø, G., Solbrække, K. N., Kristvik, E., & Werner, A. (2016). Between disruption and continuity: challenges in maintaining the ‘biographical we’ when caring for a partner with a severe, chronic illness. *Sociology of health & illness, 38*(5), 782-796. |
| SHI135 | Rhodes, P., McDonald, R., Campbell, S., Daker‐White, G., & Sanders, C. (2016). Sensemaking and the co‐production of safety: a qualitative study of primary medical care patients. *Sociology of health & illness,* *38*(2), 270-285. |
| SHI136 | Audet, M., Dumas, A., Binette, R., & Dionne, I. J. (2017). Women, weight, poverty and menopause: understanding health practices in a context of chronic disease prevention. *Sociology of health & illness, 39,* 1412–1426 |
| SHI137 | Ulfsdotter Eriksson, Y., Berg, K., Boman, U. W., & Hakeberg, M. (2017). Contract care in dentistry: sense‐making of the concept and in practice when multiple institutional logics are at play. *Sociology of health & illness, 39*(7), 1035–1049. |
| SHI138 | Lacey, S. (2017). Death in the clinic: women's perceptions and experiences of discarding supernumerary IVF embryos. *Sociology of health & illness, 39*(3), 397-411. |
| SHI139 | Moloney, M. E. (2017). ‘Sometimes, it's easier to write the prescription’: physician and patient accounts of the reluctant medicalisation of sleeplessness. *Sociology of health & illness, 39*(3), 333-348. |
| SHI140 | Noury, M., & Lopez, J. (2017). Nanomedicine and personalised medicine: understanding the personalisation of health care in the molecular era. *Sociology of health & illness, 39*(4), 547-565. |
