# Supplementary material for: Characterising and justifying sample size sufficiency in interview-based studies: systematic analysis of qualitative health research over a 15-year period
Source: BMC Med Res Methodol. 2018 Nov 21;18:148. doi: 10.1186/s12874-018-0594-7 (PMC6249736; doi:10.1186/s12874-018-0594-7)
Supplement: Supplementary file 3 — Data Extraction Form. (DOCX 15 kb) [file 12874_2018_594_MOESM3_ESM.docx]

# Additional File 3: Data Extraction Form

| **PAPER CODE*** |  |
| --- | --- |

| **Article Details** | | | |
| --- | --- | --- | --- |
| **Author(s)** |  | | |
| **Title** |  | | |
| **Journal** |  | | |
| **Year of Publication** |  | | |
| **Volume(Issue) pp.** |  | | |
| **Reviewer(s)** | 1. | 2. | 3. |
| **Date of review** | 1. | 2. | 3. |
| **Eligibility of study** | YES | NO | Discuss with colleagues |

| **Aims, Sample size & justification, participants’ characteristics, sampling technique** | |
| --- | --- |
| ***Extraction Items*** | ***Details*** |
| **Aim(s) of study** |  |
| **Setting** |  |
| **Number of interviews (participants)** |  |
| **Response rate** |  |
| **Justification for sample size** |  |
| **Participants’ characteristics** |  |
| **Inclusion/exclusion criteria** |  |
| **Sampling technique** |  |
| **Other observations, justifications related to sample** |  |
| **Country(ies)/area of data collection** |  |
| **Period of data** **collection** |  |

| **Type of analysis (IPA, thematic), Epistemological framework, Software** | |
| --- | --- |
| ***Extraction item*** | ***Details*** |
| **Method of data collection** |  |
| **Duration of interviews** |  |
| **Type of analysis** |  |
| **How many researchers analysed the data** |  |
| **Epistemological framework** |  |
| **Software** |  |
| **Other observations** |  |

**Note:** Any text within “” is copied directly from the article.
