# Supplementary material for: Characterising and justifying sample size sufficiency in interview-based studies: systematic analysis of qualitative health research over a 15-year period
Source: BMC Med Res Methodol. 2018 Nov 21;18:148. doi: 10.1186/s12874-018-0594-7 (PMC6249736; doi:10.1186/s12874-018-0594-7)
Supplement: Supplementary file 4 — Citations used by articles to support their position on saturation. (DOCX 14 kb) [file 12874_2018_594_MOESM4_ESM.docx]

**Additional File 4**

Citations used by articles to support their position on saturation

Citation used by **BMJ** article:

- Pope, C., Ziebland, S., Mays, N. (2000). Qualitative research in health care: Analysing qualitative data. *BMJ, 320*, 114-6. (BMJ18)

Citations used by **BJHP** articles:

Chamberlain, K. (1999). Using grounded theory in health psychology. In M. Murray & K. Chamberlain (Eds.), *Qualitative health psychology*. London: Sage. (BJHP05)

Dey, I. (1999). *Grounding grounded theory.* San Diego: Academic Press. (BJHP16)

Glaser, B. G., & Strauss, A. L. (1967). *The discovery of grounded theory: Strategies for qualitative research.* Chicago: Aldine Press. (BJHP16)

Marshall, N. M. (1996). Sampling for qualitative research. *Family Practice, 13*(6), 522–525. (BJHP17)

Patton, M. Q. (2002). *Qualitative research & evaluation methods, 3rd ed*. Thousand Oaks, CA: Sage. (BJHP28)

Corbin, J., & Strauss, A. (2008). *Basics of qualitative research (3rd ed.).* London, UK: Sage (BJHP30)

Creswell, J. W. (2007). *Qualitative inquiry and research design: Choosing among five approaches (2nd ed.).* London, UK: Sage (BJHP30)

Hood, J. C. (2007). Orthodoxy vs. power: The defining traits of grounded theory. In A. Bryant & K. Charmaz (Eds.), *The sage handbook of grounded theory* (pp. 151–164). London, UK: Sage (BJHP30)

Strauss, A., & Corbin, J. (1994). Grounded theory methodology: An overview. In N. K. Denzin & Y. S. Lincoln (Eds.), *Handbook of qualitative research* (pp. 273–285). London, UK: Sage (BJHP30)

Morse, J. M. (1995). The significance of saturation. *Qualitative Health Research, 5*(2), 147–149. (BJHP31; BJHP32)

Guest, G., Bunce, A., & Johnson, L. (2006). How many interviews are enough? An experiment with data saturation and variability. *Field Methods, 18*(1), 59–82. (BJHP46; BJHP50)

Mays, N., & Pope, C. (1995). Qualitative research: Rigour and qualitative research. *BMJ, 311*, 109–112. (BJHP46)

Clarke, V., & Braun, V. (2013). *Successful qualitative research: A practical guide for beginners.* London: Sage. (BJHP50)

Fugard, A., & Potts, H. (2014). *Sample size determination for thematic analysis and related qualitative methodologies: a quantitative model*. Paper presented at the 6th ESRC: Research Methods Festival, St Catherine’s College, Oxford, UK. (BJHP50)

Glaser, B. G., & Strauss, A. L. (2009). The discovery of grounded theory: Strategies for qualitative research. Piscataway, NJ: Transaction Publishers. (BJHP52)

Citations used by **SHI** articles:

Burgess, R.G. (1982) Keeping field notes. In Burgess, R.G. (ed) *Field Research: a Sourcebook and Field Manual*. London: Unwin Hyman. (SHI04)

Strauss, A.L. (1987) *Qualitative Analysis for Social Scientists*. Cambridge: Cambridge University Press. (SHI13)

Strauss, A. and Corbin, J. (1990). Basics of Qualitative Research: Grounded Theory Procedures and Techniques. Newbury Park, CA: Sage. (SHI32)

Morse, S. (2000) Determining sample size. *Qualitative Health Research, 10*, 1, 3–5. (SHI73)

Bryman, A. (2001) *Social Science Research Methods.* Oxford: Oxford University Press. (SHI78)

Polit, D. and Beck, C. (2008) *Nursing Research. 8th edn*. Philadelphia: Lippinscott, Williams and Wilkins. (SHI113)

Lincoln, Y. and Guba, E.G. (1985) Naturalistic Inquiry. Newbury Park: Sage (SHI115)
